# Procedural Challenges in Soil Sample Preparation for Pharmaceuticals Analysis

**DOI:** 10.3390/molecules30234660

**Published:** 2025-12-04

**Authors:** Agnieszka Fiszka Borzyszkowska, Ewa Olkowska, Lidia Wolska

**Affiliations:** Department of Environmental Toxicology, Faculty of Health Sciences with Institute of Maritime and Tropical Medicine, Medical University of Gdansk, Debowa 23 A, 80-204 Gdansk, Poland

**Keywords:** pharmaceuticals, extraction technique, ultrasonication, filtration, sorption, soil, LC-MS/MS

## Abstract

The determination of trace levels of pharmaceuticals in complex environmental samples requires an appropriate sample preparation stage prior to final determination. Currently, special attention should be paid to pharmaceuticals extensively used on industrial farms due to their possible emission into the soil environment, which has become a public health concern. The greatest challenge in such sample analyses lies in selecting the appropriate analyte extraction procedure. To address these challenges, five sample preparation procedures were evaluated for the determination of 24 pharmaceuticals, aiming to compare analyte recovery. We focused on commonly used stages of the procedure, such as ultrasonication and filtration, to minimize potential analyte loss. Our results indicate that some compounds can be eliminated even up to 100% (including amoxicillin, ampicillin, and lincomycin) during ultrasonication and/or filtration. The selected procedure allowed for the improvement of the recovery value in the case of 15 pharmaceuticals in comparison with the primary method. Consequently, the proposed procedure was applied to analyze environmental soil samples from the area surrounding a poultry farm. This study demonstrates that even problematic compounds, despite their low recovery, can be analyzed by the addition of isotopically labelled internal standards with acceptable accuracy. This finding is particularly important for environmental monitoring, where trace-level detection of pharmaceutical residues is often challenged by matrix complexity and analyte instability.

## 1. Introduction

Industrial poultry farming has detrimental effects on both the environment and human health [1]. Notably, it contributes to a variety of harmful compounds (including pharmaceuticals) emissions and leads to the contamination of soil, air, and surface water [2]. These activities pose significant epidemiological risks, affecting ecosystems and the organisms that inhabit them, and contribute to a decline in biodiversity. Moreover, pharmaceuticals cause risk of unknown effects on ecosystems and human health [3,4]. In order to evaluate the impacts of pharmaceuticals causing multiple problems described by Gworek et al. [5] (accumulation in the soil and transfer in the trophic chain from plants to humans, antimicrobial resistance, etc.), it is necessary to determine their concentration levels in environmental matrices. Among a variety of matrices, soil samples cause the greatest analytical difficulties [6,7]. The sample preparation stage, as analytical experts highlight, is the most important in the analytical procedure to receive accurate and precise results. Challenges during preparation of complex environmental samples (especially solid ones) to determine trace levels of pharmaceuticals are mainly caused by their various chemical structures and differences in physicochemical properties, including stability [6]. To address those aspects, many analytical protocols were proposed involving such stages as, e.g., sonication, filtration, and addition of isotopically labelled internal standards (ILIS). However, the impact of involved operations on analyte recovery and their stability is not fully investigated and evaluated [6,8]. Currently, the ultrasound-assisted extraction technique is commonly used for the analysis of organic contaminants from solid samples, which is recognized with many advantages, including simple application to a variety of matrices and possibilities to use a wide range of solvents [9]. This method has achieved widespread recognition and is now considered a standardized approach for enhancing the efficiency of the extraction process [10,11].

The analysis of compounds in the soil matrix is exceptionally difficult due to the complexity of the phenomena occurring at the interface of water—sediment. Therefore, we should generally consider “sorption” processes, which involve both surface absorption (adsorption) and volume absorption (absorption) by a solid phase (sorbent). In this context, the specific mechanisms of these processes are not distinguished [12]. Consequently, in the analysis of the soil matrix, it is necessary to consider that the same compounds can behave differently when they occur in the water and other ions. They can be adsorbed on the sediment surface, or they are incorporated into the sediment structure [13]. When pharmaceuticals are sorbed on a porous matrix-like soil, they are protected from light, but also conditions below the surface of the soil are more stable, and degradation could be limited [14]. Additionally, the bioavailability of a given compound is also strongly dependent on the sorption strength [15].

In relation to the phenomena described earlier, various factors influence the equilibrium sorption of contaminants to the soil matrix, which are related to the compound’s properties, soil characteristics, and solution characteristics [3]. Firstly, the hydrophobicity of an organic compound is determined by the octanol–water partition coefficient (LogP), evaluating the sorption properties of the non-ionizable compounds, where a higher value indicates the easier sorption capacity on hydrophobic surfaces [16]. However, the relation between the hydrophobicity of the compounds and their sorption is very complex due to the influence of other factors, e.g., soil composition and pH [17]. Secondly, the analytes’ ionization significantly affects their sorption capacity to soils, causing many interactions with the soil, including cation exchange, cation bridging, and complex formation [18]. It was found that cationic species are expected to have stronger sorption availability than other forms [19]. Finally, electrostatic interactions (including cation exchange, electrostatic attraction and repulsion), surface complexation, π-π and hydrophobic interactions, and hydrogen bonding were also reported as mechanisms influencing sorption processes [20,21].

The choice of proper procedure for pharmaceuticals’ extraction from environmental samples depends on many factors; mainly, one should consider the aim of the research, the analyte concentrations, the extraction efficiency, as well as the sensitivity and selectivity of the final measurement method [8]. The measurement of pharmaceutical concentration in soil samples is additionally related to extra challenges due to physicochemical soil properties, which have an impact on the interactions between ionized pharmaceuticals and the charged surfaces of minerals [17,22]. Among parameters influencing recovery yields are the content of organic and inorganic matter, pH, ionic strength, the type of divalent cations present and the type of minerals. Their equilibrium adjustment causes a change in the recovery rates of extraction [23]. On the other hand, many organic pollutants are able to be degraded under specific conditions, e.g., by ultrasound. The process of sonication is effective in micropollutant degradation up to this level, and can be considered a green and advanced technique for their removal to eliminate them from the aquatic environment [24,25].

Due to aforementioned reasons, it is of high importance to investigate deeply the influence of all processes applied to the extraction procedure to avoid possible analyte reduction. Pharmaceutical degradation can be caused by different agents, such as ultrasound or filtration. Our study aims to analyze the concentration of 24 selected pharmaceutical compounds from soil samples using a simple technique to reduce the loss of analytes during the extraction steps. According to the literature, analyzed compounds behave in a particular way when exposed to ultrasound. In Table S1, the summary of experiments aiming at the removal of studied pharmaceuticals, where ultrasounds were applied as the main process or as one of the assisting processes in their degradation [23,24,26,27,28,29,30,31,32,33,34,35,36,37,38,39,40,41,42,43,44,45,46]. We can clearly observe that sonification caused partial or total degradation of most mentioned compounds (14 compounds among the selected 24 compounds) under specific conditions. Their susceptibility to ultrasonic treatment was evaluated under varying conditions, including initial concentration, pH, ultrasound power, and reaction time. Consequently, it is not possible to determine which compound is the most prone to degradation; however, some exhibited exceptionally high degradation rates. Especially, ampicillin (AMP), sulfacetamide (S-ACET), CAF (caffeine) and sulfamethazine (S-META) were degraded by over 90% with the use of the optimized system described by the specific work [28,34,38,43]. Two compounds, propranolol (PROP) and sulfamethoxazole (S-ZOL), have been recognized as recalcitrant to the ultrasonic treatment, due to the fact that less than 3% was degraded under the examined conditions [33,45]. Until now, ultrasonic degradation was not evaluated for nine compounds (enrofloxacin (ENRO), lincomycin (LINC), nimesulide (NIM), sulfanilamide (S-AMID), metoclopramide (MCLO), metoprolol (MET), sulfacarbamide (S-CARB), sulfaguanidine (S-GUA), sulfathiazole (S-THIAZOL)), and in this study, it was performed for the first time. However, some previous studies performed their degradation by other techniques [31,32,39,40,47,48,49].

Furthermore, in the analytical procedures of pharmaceutical determination in environmental samples, appropriate ILIS can be used to overcome matrix effects, compensate for losses during the extraction procedure, and allow the adaptation of instrument variability, causing an improvement in data quality [50]. In accordance with the assumption that ILIS behaves in the same way as the analyte during the extraction procedure, it is very important to follow the same conditions as the analytes are present in the environmental samples. It should be performed in order to maintain the same possibility of binding to the surface of soil particles [51]. Therefore, this assumption was checked for specific ILIS in the course of this research.

The objective of this research was to improve the procedure of the extraction method to enhance the recovery of selected veterinary pharmaceuticals, which occurred in soil originating from the area of the poultry farm and agricultural fields fertilized with animal manure. This study introduces a systematic approach to developing the best practices for determining selected analytes, which are often underestimated in routine workflows and have not been comprehensively addressed in previous works. In the scope of this study, the effect of extraction processes was examined to highlight the main processes responsible for analyte loss during common steps, such as filtration and ultrasonication. The novelty of this research lies in the main key aspects: focus on proper addition of ILIS addition strategy, assessment of ultrasonication and filtration effects on the loss of analytes and significant improvements in extraction efficiency. The optimized procedure achieved measurable improvements for a substantial portion of the analytes (16 out of 36) compared with the primary method. This comprehensive evaluation establishes best practices that enhance accuracy and reliability, contributing to methodological advancements in analytical chemistry.

## 2. Results and Discussion

**Figure 1:**
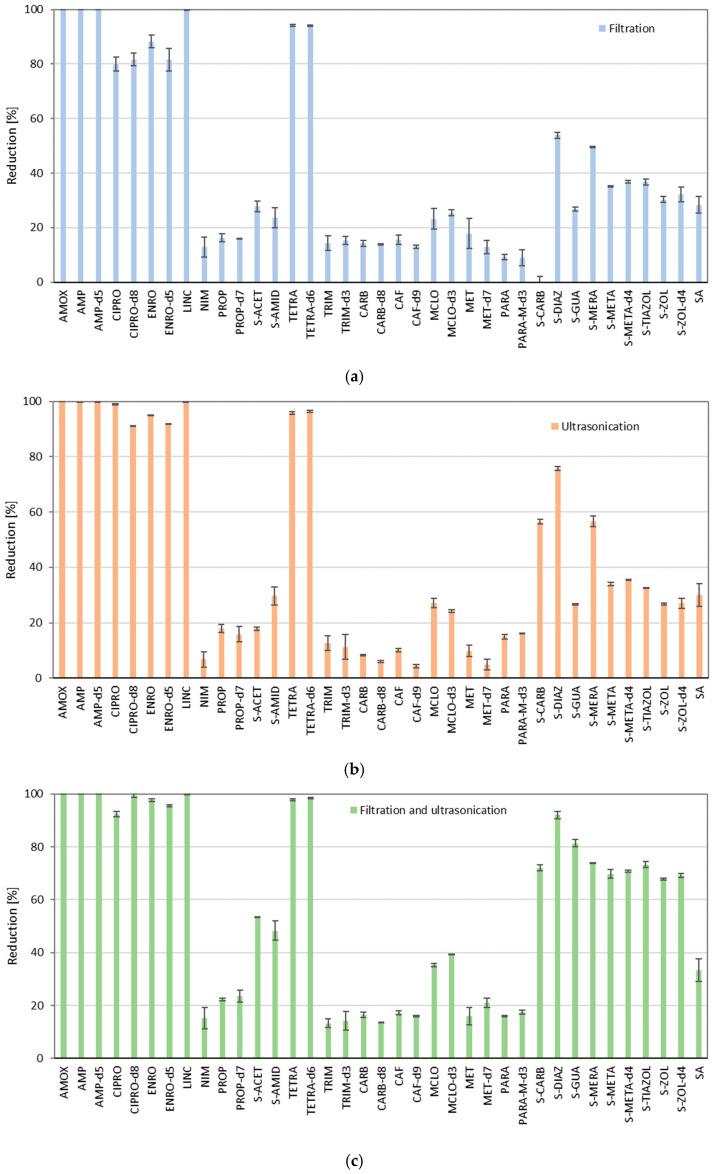
The reduction in analyte concentration during (**a**) filtration, (**b**) ultrasonication and (**c**) both of these operations.

In the present study, we aimed to find the operations in which a high loss of analytes during the extraction procedure is observed. Therefore, we have checked the influence of commonly used operations in extraction procedures, ultrasounds, filtration, or both of these processes on the stability of analytes. For this reason, simple tests determining the degradation of analytes were performed, where each step was performed according to the extraction procedure, but without the addition of soil. Results of analyte reduction caused by these processes are presented in Figure 1. It is clearly observed that the examined processes have a significant impact on the stability of the analyzed compounds. Especially for six compounds, AMOX, AMP, AMP-d5, LINC, TETRA and TETRA-d6, the reduction in their concentration during tests determining the degradation of analytes by filtration, ultrasonication, and both of them reached almost 100%. Then CIPRO (ciprofloxacin), CIPRO-d8, ENRO, and ENRO-d5 were degraded at relatively high levels, and filtration caused 80–88% of degradation, ultrasonication caused 91–95% of degradation, while both of these processes caused 96–99% of the degradation. Due to this fact, the extraction procedure should be simplified, especially to avoid loss of the aforementioned analytes.

Additionally, degradation tests of analytes applying ultrasonication and filtration processes allowed us to determine their stability properties. In this regard, we observed that NIM, PROP, PROP-d7, TRIM (trimethoprim), TRIM-d3, CARB (carbamazepine), CARB-d8, CAF, CAF-d9, MET, MET-d9, PARA (paracetamol) and PARA-M-d3 were the most stable among the examined compounds, and their reduction during ultrasonication and filtration was the lowest and did not exceed 20% of degradation. Up to that, we can observe that pharmaceutical compounds and their respective ILIS have a similar susceptibility to the examined processes, which is important in using them in the analysis of environmental samples. Therefore, the addition of deuterated standards to samples before the extraction procedure reflects the behaviour of analytes and significantly simplifies calculations as well as improves the reliability of results.

To understand the phenomenon of relatively high reduction by ultrasound treatment, we first focused on compounds with the highest water solubility: MET, LINC, CIPRO, CAF and AMP with respective solubilities of 1000, 50, 30, 21.6, and 10.1 g L^−1^. Among these, LINC was completely degraded by ultrasound, while MET, CIPRO, CAF and AMP were degraded by 9.8, 80, 21.6 and 99.7%, respectively. This suggests that high solubility may be associated with greater susceptibility to sonochemical degradation. However, it also depends on the molecular structure, as demonstrated by MET and CAF, which remained relatively resistant to ultrasound treatment. Previous studies on the sonochemical degradation of those pharmaceuticals are described in detail in Table S1, although for MET and LINC, sonochemical treatment was applied for the first time in the scope of this work. For instance, research by Montoya−Rodríguez found that AMP was completely degraded by ultrasonic treatment under optimized conditions [28]. However, CAF was removed by 21.6% by the ultrasound treatment in our experimental conditions, while the work of Ziylan−Yavas shows that this compound was easily decomposed by ultrasound and 1 h of single ultrasonic process allowed for more than 90% of CAF degradation [38]. Therefore, solubility is an important but not exclusive determinant of sonochemical degradation.

Other factors which can influence sonochemical degradation are molecular weight, octanol-water partition coefficient (LogP), pKa and size of molecules [25,52]. To examine the dependency of those factors, appropriate graphs are prepared based on pKa and LogP values found in literature (Figure S3) [53,54,55]. Nanzai et al. [49] reported that more hydrophobic monocyclic aromatic compounds degrade faster because of their tendency to accumulate at the bubble–water interface. In the present study, due to the wide diversity of chemical structures, a similar trend was not observed. The research team of Xiao et al. [50] expected a correlation whereby smaller molecules should degrade faster due to their faster diffusion to the interfacial region of cavitation bubbles. However, experimental studies suggested that large compounds were more effectively degraded by continuous-wave ultrasounds. In our study, compounds with the largest molecular masses, i.e., TETRA, LINC, AMOX, ENRO, AMP, and CIPRO (with molecular weights of 444.4, 406.5, 365.4, 359.4, 349.4, and 331.34, respectively), showed the highest degradation by ultrasound in more than 95%.

**Figure 2:**
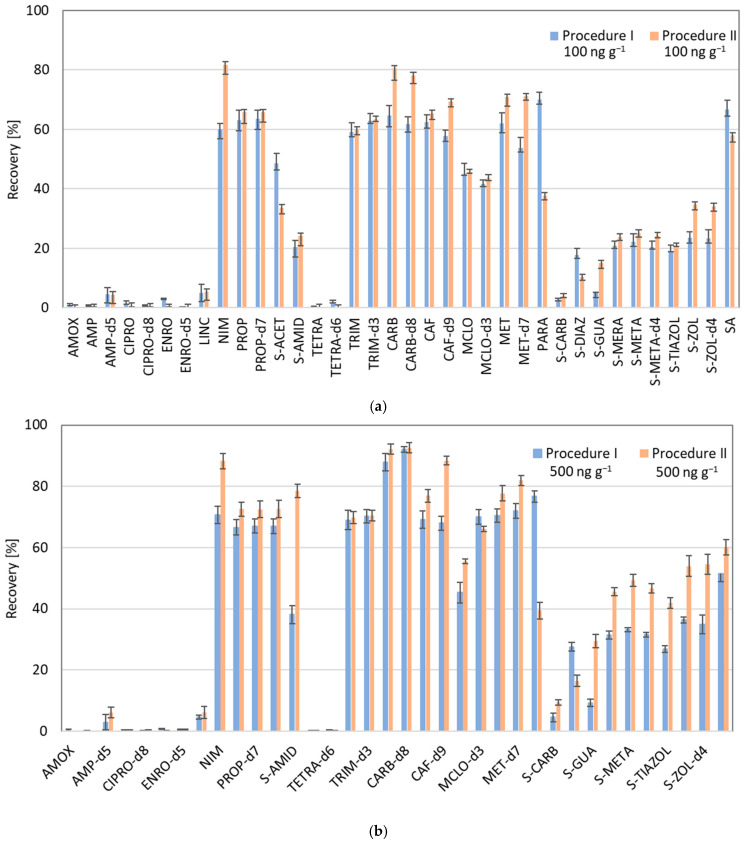
Comparison of analytes’ recovery from the experiments using dry (Procedure I) and wet soil (Procedure II) for the dose of standards 100 ng g^−1^ (**a**) and 500 ng g^−1^ (**b**).

Then, the recovery tests from soil samples were performed according to the extraction methodology described in the procedure proposed by Golovko et al. (2016), with modifications considering the stability of pharmaceuticals and the use of ILIS under ultrasound conditions, and with filtration before final analysis [7]. In the initial phase of our study, two procedures were compared, for two levels of standards added to the wet and dry soil, 100 ng g^−1^ and 500 ng g^−1^. Results from these experiments are presented in Figure 2a,b.

For most of the extracted compounds, higher recovery was obtained for the procedure where standards were added to the wet soil for both examined doses. The ratio between recovery obtained using procedure I and II was calculated for compounds with recovery of extraction higher than 5%, and for the lower dose of 100 ng g^−1^ of soil, extraction from wet soil was more efficient for 23 compounds (LINC, NIM, PROP, PROP-d7, S-AMID, TRIM, TRIM-d3, CARB, CARB-d8, CAF, CAF-d9, MCLO, MCLO-d3, MET, MET-d7, S-CARB, S-GUA, S-MERA, S-META, S-META-d4, S-THIAZOL, S-ZOL and S-ZOL-d4), while less efficient for four compounds (S-ACET, PARA, S-DIAZ and SA). A similar trend was observed for the higher dose of standards (500 ng g^−1^), except for two compounds, which behaved differently than using a lower dose; MCLO-d3—which was extracted more efficiently by enrichment in dry soil, and SA—which was extracted more efficiently by enrichment in wet soil. It was noticed that 11 of all analyzed compounds were extracted with a recovery efficiency lower than 10% at the lower dose of standards. Due to the environmentally relevant concentrations, which are relatively low, in the further steps of the research, only the lower levels of analytes were studied.

**Figure 3:**
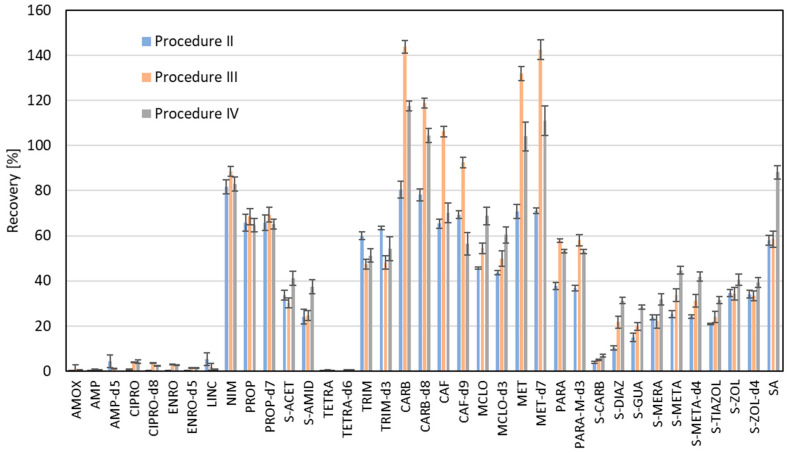
Comparison of recovery values obtained from experiments before the modification (Procedure II), with omission only filtration (Procedure III), and omission of ultrasonication and filtration (Procedure IV).

Afterwards, experiments with omission of filtration (Procedure III) and omission of ultrasonication (Procedure IV) steps were performed to study their impact on recovery efficiency. The results of these measurements are shown in Figure 3. We observed that the most significant contributions in raising analytes recovery are caused by omitting filtration for four pairs of standards and their respective ILIS: CARB and CARB-d8, CAF and CAF-d9, MET and MET-d7, PARA and PARA-M-d3. However, we must realize that this simplification of the procedure was intended to raise the recovery for the compounds, which revealed the lowest efficiency in the primary experiments. This assumption was fulfilled for four pairs of analytes (CIPRO, ENRO, TETRA and S-CARB) and their respective ILIS, which were recovered below 10% in the primary test. For CIPRO, ENRO, and their respective ILIS, the effectiveness of simplified procedures increased about 10 times. The effectiveness of TETRA extraction was still at a very low level in the simplified procedures, but these results were repeatable. However, the extraction of LINC was decreased due to the simplification of the procedure, while for AMOX and AMP, the recovery changes were not observed. It is worth noting that compounds from the group of derivatives of sulfanilamide (with the group of 4-aminobenzenesulfonamide) behaved similarly, with the rise in recovery effectiveness by omitting filtration and even higher recovery effectiveness by omitting both of these processes. The highest increase in recovery up to two times was observed for S-DIAZ with the omission of filtration, while omitting both filtration and ultrasonication caused a rise in the recovery efficiency up to three times.

**Figure 4:**
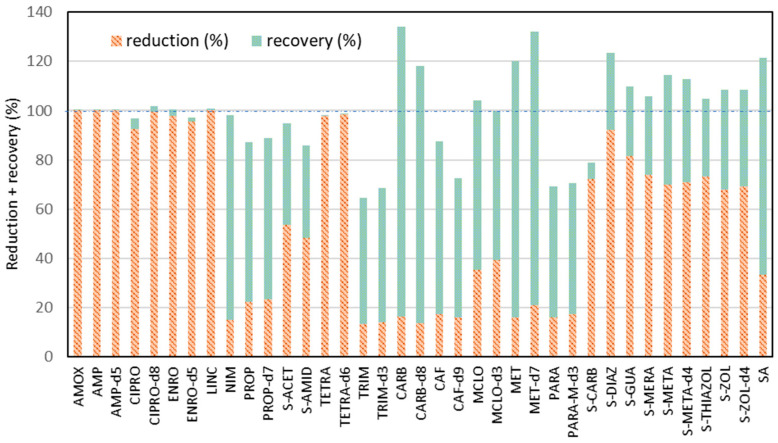
The reduction in analyte concentration during ultrasonication and filtration processes in relation to their recovery values using the extraction procedure, omitting both of these processes (Procedure IV).

Considering the fact that some of the measured analytes are reduced significantly during ultrasonication and filtration processes, it turns out that it is necessary to compare the final efficiency of recovery resulting from Procedure IV with the loss of analytes resulting from the experiment of the analytes’ reduction during ultrasonication and filtration. For this purpose, a summary is prepared in Figure 4, where both values of recovery and reduction have been added into one column for each analyte. It was observed that for most of the analyzed compounds, the total values of recovery and reduction were over 95%, while for 10 compounds, this value was lower than 95%. The lowest values (approx. 65–71%) were noticed for pairs of compounds: TRIM—TRIM-d3 and PARA—PARA-M-d3.

In the work of Lin et al. (2011), it was demonstrated that trimethoprim exhibits moderate to strong sorption among examined anti-inflammatory drugs and antibiotics for sorption and degradation ability in soils [56]. The sorption of acetaminophen was examined by Lin et al. (2010), they suggested that chemical sorption may be the mechanism of sorption of this compound onto sediments; therefore, the desorption process was very weak [57]. Our study indicates that under the selected extraction conditions, the affinity of these compounds to the soil/minerals was the highest, and some of these analytes were not desorbed from the soil surface. Described experiments showed that the ultrasonically process and filtration during the extraction step causes the reduction in recovery efficiency of most of the investigated analytes.

**Figure 5:**
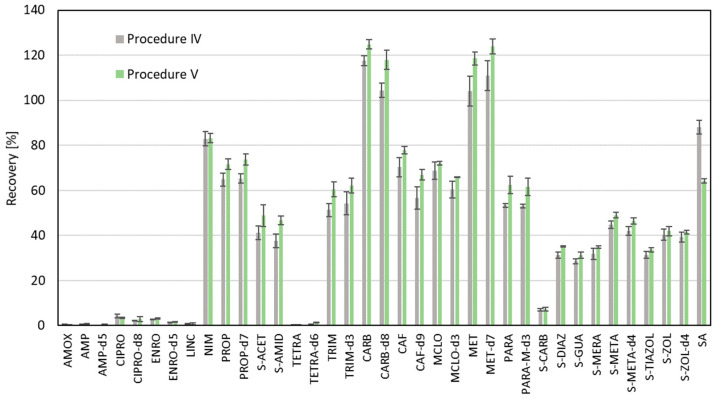
Comparison of recovery values of the experiment with omission of filtration and ultrasonication steps in the extraction step (Procedure IV) with the experiment where ultrasonication was applied after the reconstitution step (Procedure V).

In the next steps of the optimal procedure, we observed that after evaporation of solvents, the solid residue was formed inside the vials. During the reconstitution step, when 1 mL of methanol was added, turbulent shaking was applied. However, at the bottom of the vial, there was still an undissolved residue. In order to disperse this residue, an additional 5 min of ultrasonication to homogenize the solution was applied (Procedure V). In Figure 5, a comparison of Procedure IV (omitting filtration and ultrasonication) and Procedure V is presented. For most of the analytes, the higher efficiency of recovery was noticed, instead of SA and CIPRO. The recovery efficiency for the other analytes increased by approx. 4%. It can be concluded that Procedure V was the most appropriate. 

**Figure 6:**
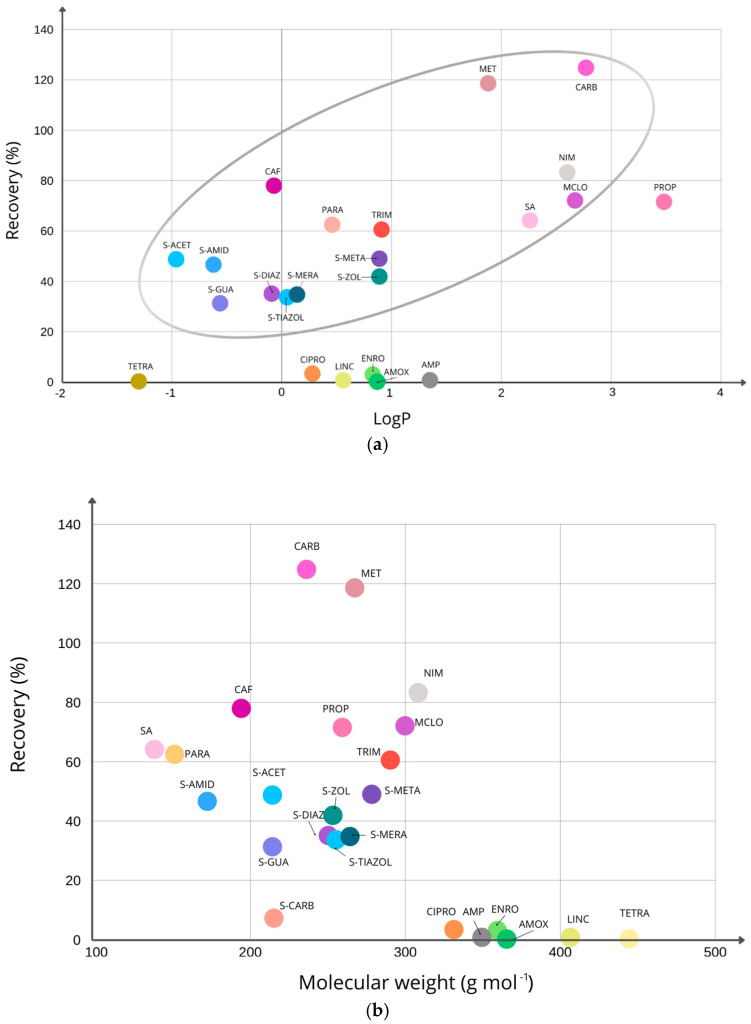
The comparison of the recovery values for each analyzed compound (obtained with the use of procedure V, which are marked with dots on the graph) with LogP values (**a**) and molecular weight (**b**).

With the aim of gaining a deeper understanding of the phenomena that can cause problematic challenges resulting in significant loss of analytes during the extraction process, it was decided to interpret deeper based on their chemical properties. Therefore, a summary of the basic values, including molecular weight, pKa, LogP and water solubility, is summarized in Table S3. To better illustrate the dependencies between experimental recovery for 25 target compounds and the corresponding LogP values, the plot of this relation is prepared (Figure 6a). The higher value of LogP should indicate the easier sorption capacity on hydrophobic surfaces; therefore, we expected the following relation: the higher LogP value is connected with the higher recovery value [58]. We observed that the experimental data obtained for some compounds deviated considerably from this assumption, and similarly, as in previous studies, some deviations occurred [59]. This deviation was observed for compounds with the lowest recovery: CIPRO, LINC, ENRO, AMOX, and AMP. On the other hand, we observed that compounds from the sulfonamide group (S-AMID, A-ACET, S-GUA, S-DIAZ, S-THIAZOL and S-MERA) behaved similarly and comparable recovery levels for them were obtained. For the compounds (MCLO, NIM, PROP, CARB, and MET) with the higher LogP, the described relation was observed, and it was favourable for the higher efficiency of the recovery (more than 70%).

Then, the relation between the molecular weight of pharmaceuticals and their availability to recover from soil is examined (Figure 6b). Greater molecules (with the molecular weight higher than 330 g mol^−1,^ including CIPRO, AMP, ENRO, AMOX, LINC, and TETRA) were difficult to extract from the soil, and the recovery values were below 5%. Aouant et al. (2025) in the review about extraction for pharmaceuticals in sediments, suggested that compounds with a higher molecular weight might have a lower predisposition to migrate into the sediment matrix [3]. Moreover, for these compounds, the expected relation of recovery with LogP (Figure 6a) was not observed.

For the comparison of the significance of the recovery differences between the studied procedures for each compound, the *t*-test was performed. Detailed results from these calculations are presented in Table S4. Additionally, for the whole overview of studied procedures, diagrams of the recovery for each separate analyte are shown. This calculation allowed us to determine the significance of the dependence between the recovery of each analyte and the use of the examined conditions. The results with the significance level of *p* parameter below 0.05 are marked (bold values), and special attention should be paid to these compounds, because the impact of procedure conditions on analytes’ recovery is confirmed. The high significance of any tested procedure was found for 23 compounds among 36 studied compounds (including ILIS). The most frequently noted significant difference was found between Procedure I and other procedures, which means that efforts aiming to improve the primary procedure were successful. Especially, it is very visible for S-AMID, MCLO, MCLO-d3, MET, MET-d7, PARA, S-DIAZ, S-GUA, S-MERA, S-META, S-META-d4, S-THIAZOL, S-ZOL and S-ZOL-d4, where each modification of procedures caused the desirable effect and improved recovery efficiency.

Taking into account the most problematic analytes, with lower efficiency than 10% (AMOX, AMP, AMP-d5, CIPRO, CIPRO-d8, ENRO, ENRO-d5, LINC, TETRA, TETRA-d6, S-CARB), it can be emphasized that for analytes ENRO, ENRO-d5, TETRA-d6 and S-CARB, the recovery efficiency obtained after using Procedure V was the highest among the studied procedures. Additionally, it is worth mentioning that Procedure V demonstrated practical advantages in environmental sample detection. Energy consumption was reduced by omitting the ultrasonic treatment step, replacing it with shaking, which maintained a comparable processing time. The amount of reagents used remained the same as in other procedures, ensuring no additional chemical cost. These factors highlight Procedure V as a more energy-efficient and equally time-effective alternative without compromising analytical performance. However, in the case of AMP-d5 and LINC, we observed the opposite effect, and the highest efficiency was observed for Procedure II, but the difference is not significant according to *t*-test calculations. For the above-mentioned analytes, the efforts aiming for their desorption from the soil surface should be further studied. It is worth emphasizing that each procedure caused a repeatable degree of recovery and was comparable with the respective ILIS. Therefore, the calculations of analyte concentration were made based on the respective calibration curves in the next step of the research.

**Figure 7:**
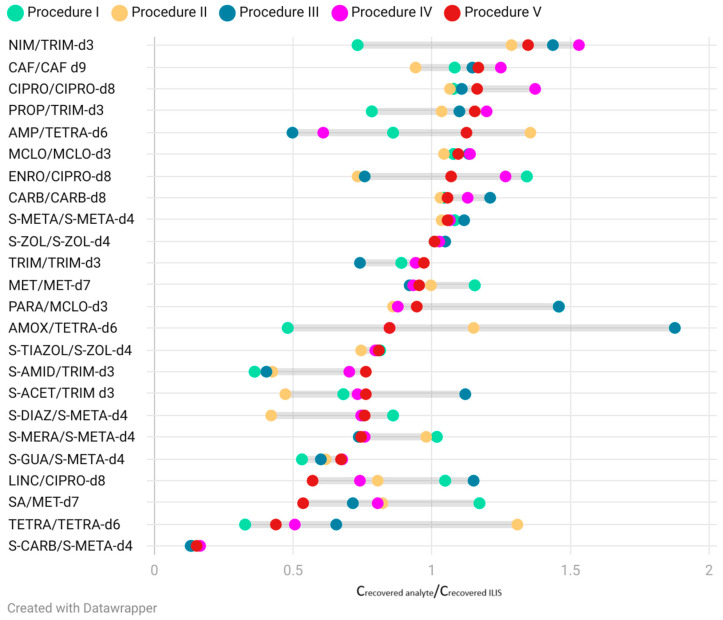
Comparison of the ratio of C_recovered analyte_/C_recovered ILIS_ for each procedure.

At the last stage of the presented study, the calculation of pharmaceutical concentration was performed according to the calibration curve using ILIS (spiked together with analytes before extraction). Then, the obtained concentrations were divided by the concentrations of the reference sample, which consisted of the blank solution of analytes and ILIS with the same concentration as spiked into the soil [60]. These values should be close to 1 for each analyzed compound and with each procedure. These results of the ratio C_recovered analyte_/C_recovered ILIS_ for each analyzed pharmaceutical are summarized in Figure 7. Ratios, obtained according to the results from Procedure V, are ordered from the highest to the lowest value. In our study, these compounds, which had deuterated equivalents as ILIS and aforementioned high recovery values, e.g., MCLO, CARB, S-META, S-ZOL, TRIM and MET, were characterized by the ratio C_recovered analyte_/C_recovered ILIS_ close to 1 and were similar for each procedure. On the other hand, these compounds, which were characterized by low efficiency of recovery, e.g., CAF, CIPRO, TETRA, despite the fact that the deuterated equivalent was taken into calculations, differentiated values were noted. The greatest variation in values was observed for TETRA, but the recovery of this compound and their deuterated equivalent was below 1%, and at this level, deviations can be explained by an error in chromatographic analysis or extraction procedure. A relatively low recovery rate may result from strong or specific sorption of TETRA to the soil samples [61]. Then we were focused on these compounds, which have no available deuterated equivalent, and similar conclusions were found, that the ratio C_recovered analyte_/C_recovered ILIS_ can be similar and close to 1 for these compounds with a relatively high value of recovery. Although we also observed that for S-GUA and S-CARB, where S-META-d4 was used as a standard, this ratio was relatively low but repeatable for each procedure. In those cases, for the determination of the mentioned analytes in the environmental samples, compensation and superior performance of our method can be achieved by the use of predetermined recovery factors [62].

According to the recommendations, the concentrations in environmental samples could be corrected by a recovery factor, in such cases when recoveries are outside 80–120% [63]. In our study, we decided to use the recovery factor for calculation of each analyte’s concentration in environmental samples. Recovery factor for each analyte’s was determined by the ratio of recovered ILIS concentration to recovered analyte concentration (C_recovered ILIS_/C_recovered analyte_) using the results obtained according to Procedure V. These values are shown for each analyte with the results of the detected pharmaceuticals concentration in Table 1. In the investigated samples, which originated from soil collected in fields fertilized with organic manure and soil from an area close to a poultry farm, five pharmaceuticals were detected above LOQ (i.e., AMOX, CIPRO, S-ACET, CAF, PARA) and up to 10 pharmaceuticals were detected above LOD (i.e., AMOX, AMP, CIPRO, S-ACET, MET, S-CARB, CAF, PARA, S-GUA, S-META). This method allowed us to confirm the analyzed pharmaceuticals in environmental soil samples.

This study enabled the identification of optimal parameters for the procedure, which significantly simplified the workflow and improved recovery rates. Nevertheless, certain compounds remain particularly challenging to recover efficiently. Future research should focus on enhancing recovery for these problematic compounds. Potential directions include testing alternative extraction solvents, optimizing shaking conditions, and exploring hybrid approaches that combine mechanical agitation with mild ultrasonic treatment or other energy-saving techniques [3]. These improvements could further increase the robustness and applicability of the procedure in environmental sample analysis. Additionally, the development of methods enabling straightforward and reliable analysis of pharmaceuticals in diverse environmental matrices will provide critical insights into their environmental fate and help to mitigate potential consequences for human health, allowing risk assessment and pollution control strategies.

## 3. Materials and Methods

### 3.1. Materials

Methanol (MeOH), acetonitrile (ACN) and isopropanol (IPA) of LC-MS grade were purchased from Supelco (Bellefonte, Pennsylvania USA), ammonia (p.a.) from Chempur (Piekary Śląskie, Poland), formic acid (FA) of 98–100%, ammonium formate with LCMS grade and acetone (99.9%) from Merck (Darmstadt, Germany). Deionized water (DI) was obtained using the Hydrolab system (Straszyn, Poland).

Most of the analyte standards and ILIS were purchased from LGC (Luckenwalde, Germany), amoxicillin (AMOX), ampicillin (AMP), ampicillin-d5 (AMP-d5), ciprofloxacin (CIPRO), ciprofloxacin-d8 (CIPRO-d8), enrofloxacin (ENRO), enrofloxacin-d5 (ENRO-d5), carbamazepine (CARB), carbamazepine-d8 (CARB-d8), caffeine (CAF), caffeine-d9 (CAF-d9), salicylic acid (SA), lincomycin (LINC), metoclopramide (MCLO), metoclopramide-d3 (MCLO-d3), metoprolol (MET), metoprolol-d7 (MET-d7), nimesulide (NIM), paracetamol (PARA), propranolol (PROP), propranolol-d7 (PROP-d7), sulfacetamide (S-ACET), sulfacarbamide (S-CARB), sulfadiazine (S-DIAZ), sulfaguanidine (S-GUA), sulfamerazine (S-MERA), sulfamethazine-d4 (S-META-d4), sulfamethoxazole (S-ZOL), sulfamethoxazole-d4 (S-ZOL), sulfathiazole (S-THIAZOL), tetracycline (TETRA), tetracycline-d6 (TETRA-d6), trimethoprim (TRIM), trimethoprim-d3 (TRIM-d3). While paracetamol-methyl-d3 (PARA-d3), sulfamethazine (S-META) and sulfanilamide (S-AMID) were purchased from Sigma-Aldrich (St. Louis, MI, USA).

### 3.2. Preparation of Standard Solutions

Standard solutions of native standards and ILIS with a concentration of 1 mg mL^−1^ in methanol were prepared. Working solutions of the selected analytes (native standards and/or internal) with concentrations of 10 µg mL^−1^ were prepared by diluting standard solutions. The soil was then enriched with a mixture of working solutions at two concentration levels, low 100 ng g^−1^ and high 500 ng g^−1^. Standard and working solutions were stored in the dark at −20 °C.

### 3.3. Extraction Methods

The detailed scheme of two procedures for determining pharmaceuticals in soil samples is given in Figure S1. Firstly, soil samples (dry or humid) were prepared by weighing 2 g of soil into 12 mL vials, wetted with acetone and dripped with pharmaceutical and internal standards solutions in MeOH. Vials (with three replicates for each dose and each procedure) were then shaken, and the liquid was allowed to evaporate at room temperature. Final concentrations of the targeted compounds and ILIS were 500 and 100 ng g^−1^ dry weight (d.w.) for each procedure. Pharmaceuticals were extracted according to the procedure of Golovko et al. (2016) with the use of two extraction steps supported by ultrasonication (50 kHz, 15 min in each step) [7]. Firstly, with 4 mL of the mixture of ACN and 0.1% aqueous solution of formic acid (1/1 *v*/*v*), and secondly with 4 mL of the mixture of ACN, IPA, and 0.1% aqueous solution of formic acid (3/3/4 *v*/*v*/*v*). The extract was filtered through syringe filters (0.22 μm, PTFE hydrophobic purchased from Labfil, China), evaporated to dryness and reconstituted with 1 mL of MeOH. Samples before final analysis by LCMS/MS techniques were centrifuged to remove solid particles.

Procedures no. III, IV, and V were based on procedure II with slight modifications. These changes in the primary procedure aimed to evaluate the effect of ultrasonication and filtration processes on the stability of analytes. The most simplified procedure (no. IV) involved shaking instead of ultrasonication in each step of extraction, and also ultrasonication after reconstitution with methanol was omitted. This procedure is presented in Figure S2, where the processes involved in procedures III and IV are also indicated. Additionally, in procedure III, filtration was applied, while ultrasonication was omitted, and in procedure V, filtration and ultrasonication in the extraction step were omitted, but after the reconstitution step, 5 min of ultrasonication was applied. The best procedure was selected based on the analyte recovery value.

To determine the initial concentration of analytes without applying any extraction process, a reference sample was prepared. This sample contained the same amount of analytes as those introduced into the soil during the extraction experiments. The pharmaceuticals were dissolved in 1 mL of methanol, ensuring that the reference represented the theoretical concentration expected in the final extract.

### 3.4. Tests of the Loss of Analytes in Ultrasonication and Filtration

The degradation of analytes by ultrasonication and filtration, and both of these processes, were checked during experiments conducted analogously to previously described procedures of extraction, but with the omission of the soil presence. Briefly, the 1 mL solution of the analytes and IS were prepared with a concentration of 200 µg L^−1^ in acetone (the total dose of analytes was the same as during the extraction procedure). To check the degradation during both processes, ultrasonication and filtration, firstly 4 mL of the mixture of ACN and 0.1% aqueous solution of formic acid (1/1 *v*/*v*) was added and sonicated for 15 min at 50 kHz, then 4 mL of the mixture of ACN, IPA, and 0.1% aqueous solution of formic acid (3/3/4 *v*/*v*/*v*) was added and sonicated for additional 15 min. Subsequently, the solution was filtered through syringe filters (0.22 μm, PTFE hydrophobic purchased from Labfil, Shaoxing, China), evaporated to dryness and reconstituted with 1 mL of MeOH. In the test, aiming to check the influence of single processes, ultrasonication or filtration was omitted accordingly. After the reconstitution step, samples were analyzed by the LCMS/MS technique.

### 3.5. Final Determination

All experiments were performed using Nexera X2 liquid chromatograph coupled with the LCMS 8050 triple quadrupole spectrometer from Shimadzu Corp (Kyoto, Japan), equipped with two pumps (LC-30AD model, Kyoto, Japan), autosampler (SiL-30AC model, Kyoto, Japan), thermostat (CTO-20AC, Kyoto, Japan) and system controller (CBM-20A, Kyoto, Japan).

The chromatographic separation was carried out on a 150 × 2.1 mm × 2.6 µm Kinetex Phenyl-Hexyl column (Phenomenex, Torrance, California, USA) using 0.1% formic acid (for the analysis in acidic mode) or ammonia buffer with pH = 8 (for the analysis in alkaline mode) (A) and methanol (B) as the mobile phase at a gradient of 5% to 85% B within 11 min.

The conditions for the mass spectrometer temperatures and flow rates were as follows: ESI-interface (300 °C), desolvation line (250 °C), heat block (350 °C), nitrogen as nebulizing gas (3 L min^−1^), drying gas (10 L min^−1^), and air heating gas (10 L min^−1^).

Analyses were performed using Multiple Reaction Monitoring (MRM) mode with the selection of optimal conditions (the optimum cone voltages and collision energies), and the details of these measurements, the most abundant product ion used for quantification is included in Table S2, as well as the indicative retention times. Additionally, two other transition ions for each compound were also noticed for our confirmation purposes.

### 3.6. Determination of Selected Pharmaceuticals in Environmental Samples

Soil samples were collected in areas of the poultry farm and agricultural fields nearby in Poland. The samples were freeze-dried and then stored in −70 °C before the extraction procedure, which was selected after examination of 5 extraction procedures. The amount of soil was analogous to the preliminary experiments. Each sample was enriched with the mixture of IS with the target dose of 100 ng g^−1^ of sample. Finally, the samples were analyzed using LCMS/MS in the MRM mode. The content of the analytes in the samples was determined using the surface areas of measured pharmaceuticals and the used IS, for the following calculations according to the obtained calibration curves.

### 3.7. Data Analysis

As part of the calibration curve validation, the linearity, limit of detection (LOD), and limit of quantification (LOQ) were determined. Linearity was determined on the basis of the prepared calibration curves for analytes. The calibration curves were in the range of 1 µg L^−1^–500 µg L^−1^, three replicates were measured for each concentration level (1 µg L^−1^; 2.5 µg L^−1^; 5 µg L^−1^; 10 µg L^−1^; 25 µg L^−1^; 50 µg L^−1^; 100 µg L^−1^; 250 µg L^−1^; 500 µg L^−1^). The calibration curves were prepared by adding working solutions of the pharmaceutical standards at specified concentrations to each calibration level, and the constant concentration of ILIS was equal to 100 µg L^−1^ instead of CARB-d8, whose concentration was equal to 50 µg L^−1^. Regression equations for each analyte were obtained using the linear regression method, with the respective internal standard (or selected according to the similarity level of recovery), and then the coefficient of determination (R^2^) was determined. For recovery results between procedures, a paired two-tailed *t*-test (α = 0.05) was performed. We used a significance level of α = 0.05. Results were considered statistically significant when *p* ≤ 0.05.

LOD and LOQ were determined according to the International Committee on Harmonization guideline based on the standard deviation of the response and the slope [64].

## 4. Conclusions

The analysis of soil samples is highly challenging due to the possibility of determining compounds’ sorption, interactions between ionized pharmaceuticals and the charged surfaces of minerals, the complexity of the soil matrix, etc. Our study has shown that the extraction conditions of pharmaceuticals from soil samples influence their efficient recovery. Some processes, frequently used in extraction procedures, can significantly reduce the recovery due to the limitations of analyte stability during ultrasound steps and their loss by filtration. Therefore, we focused on the evaluation of different sample preparation conditions (Procedure I–V) by omitting the mentioned processes and testing the stability of analytes. We observed that simplification of the procedure, on the one hand, can raise the recovery, but on the other hand, it also saves us time and effort. Procedure V (with omitting filtration and ultrasonication in the extraction step, and additional ultrasonication of reconstitution samples) allowed for the achievement of the highest recovery values for the most problematic tested pharmaceuticals (AMOX, AMP, CIPRO, ENRO, LINC, TETRA, S-CARB). This phenomenon, which allows higher recovery values—especially the previously mentioned compounds—is highly valuable as an innovative approach to examining substances with diverse physicochemical properties. The use of ILIS can effectively compensate for residual matrix effects and simplify calculations, but such compounds’ recovery factor could be applied. Finally, this study highlights the proper procedure planning to achieve the reliability and efficacy of the used method for the routine residue monitoring of pharmaceuticals in soil samples. Further investigation is needed for a deeper understanding of the interactions of pharmaceuticals with solid samples and the effect of the used extraction conditions on their stability.

## Figures and Tables

**Table 1:** The summary of pharmaceutical concentration (calculated for dry weight samples) in environmental samples, where LOD—limit of detection, LOQ—limit of quantification, SD—Standard Deviation, F1, F2—samples from fields fertilized with organic manure, A—sample from an area close to a poultry farm.

Analyzed Compound	Recovery Factor	LOD [ng g^−1^]	LOQ [ng g^−1^]	C_F1_ ± SD [ng g^−1^]	C_F2_ ± SD [ng g^−1^]	C_A_ ± SD [ng g^−1^]
AMOX	1.18	2.4	7.1	<LOD	<LOQ	138 ± 49
AMP	0.89	1.9	5.8	<LOQ	<LOQ	<LOQ
CIPRO	0.86	2.7	8.0	14.5 ± 3.6	12.5 ± 7.2	<LOQ
ENRO	0.93	2.0	6.1	<LOD	<LOD	<LOD
LINC	1.75	1.3	3.8	<LOD	<LOD	<LOD
NIM	0.74	0.26	0.78	<LOD	<LOD	<LOD
PROP	0.86	0.12	0.35	<LOD	<LOD	<LOD
S-ACET	1.31	0.31	0.93	3.57 ± 1.32	<LOD	<LOD
S-AMID	1.31	2.4	7.3	<LOD	<LOD	<LOD
TETRA	2.27	4.0	12	<LOD	<LOD	<LOD
TRIM	1.03	0.35	1.0	<LOD	<LOD	<LOD
CARB	0.94	0.28	0.84	<LOD	<LOD	<LOD
CAF	0.86	0.94	2.8	4.29 ± 1.31	<LOD	<LOD
MCLO	0.91	0.26	0.77	<LOD	<LOD	<LOD
MET	1.05	0.62	1.8	<LOQ	<LOQ	<LOQ
PARA	1.06	0.47	1.4	16.6 ± 3.3	<LOD	<LOD
S-CARB	6.46	2.2	6.7	<LOD	<LOQ	<LOD
S-DIAZ	1.32	0.52	1.6	<LOD	<LOD	<LOD
S-GUA	1.48	0.88	2.6	<LOD	<LOQ	<LOD
S-MERA	1.34	0.38	1.2	<LOD	<LOD	<LOD
S-META	0.95	0.48	1.4	<LOQ	<LOQ	<LOQ
S-TIAZOL	1.23	0.28	0.85	<LOD	<LOD	<LOD
S-ZOL	0.99	0.27	0.82	<LOD	<LOD	<LOD
SA	1.86	6.2	19	<LOD	<LOD	<LOD

## Data Availability

The datasets used and/or analyzed in the present study are available from the corresponding author upon reasonable request.

## Supplementary Materials

The following supporting information can be downloaded at: https://www.mdpi.com/article/10.3390/molecules30234660/s1, Table S1. Summary of studies aiming at the removal of selected pharmaceuticals by the ultrasonic (US) technique; Figure S1. Scheme of procedures no. I–II for determining pharmaceutical analytes in soil samples; Figure S2. Scheme of procedures no. III-V for determining pharmaceutical analytes in soil samples; Table S2. Optimized LC-MS/MS instrumental parameters for all analytes, including the isotopically labelled internal standards; Figure S3. The comparison of the pharmaceuticals’ degradation by ultrasound treatment (US) with LogP values (a), pKa values (b) and molecular weight (c); Table S3. The list of analyzed pharmaceuticals and their selected physico-chemical properties; Table S4. Performance comparison of *p*-values between studied procedures (Procedure I–V) with the *t*-tests for each examined compound. Reference [65] has been cited in the Supplementary Materials.

## Abbreviations

The following abbreviations are used in this manuscript:

ACN	acetonitrile
AMOX	amoxicillin
AMP	ampicillin
AMP-d5	ampicillin-d5
CAF	caffeine
CAF-d9	caffeine-d9
CARB	carbamazepine
CARB-d8	carbamazepine-d8
CIPRO	ciprofloxacin
CIPRO-d8	ciprofloxacin-d8
ENRO	enrofloxacin
ENRO-d5	enrofloxacin-d5
FA	formic acid
ILIS	isotopically labelled internal standards
IPA	isopropanol
LINC	lincomycin
LOD	limit of detection
LOQ	limit of quantification
LogP	octanol–water partition coefficient
LCMS/MS	Liquid Chromatography-Tandem Mass Spectrometry
MeOH	methanol
MCLO	metoclopramide
MCLO-d3	metoclopramide-d3
MET	metoprolol
MET-d7	metoprolol-d7
MRM	Multiple Reaction Monitoring
NIM	nimesulide
PARA	paracetamol
PARA-M-d3	paracetamol-methyl-d3
PROP	propranolol
PROP-d7	propranolol-d7
S-ACET	sulfacetamide
S-AMID	sulfanilamide
S-CARB	sulfacarbamide
S-DIAZ	sulfadiazine
S-GUA	sulfaguanidine
S-MERA	sulfamerazine
S-META	sulfamethazine
S-META-d4	sulfamethazine-d4
S-ZOL	sulfamethoxazole
S-ZOL-d4	sulfamethoxazole-d4
S-THIAZOL	sulfathiazole
SA	salicylic acid
TETRA	tetracycline
TETRA-d6	tetracycline-d6
TRIM	trimethoprim
TRIM-d3	trimethoprim-d3
