# Supplementary material for: Procedural Challenges in Soil Sample Preparation for Pharmaceuticals Analysis
Source: Molecules. 2025 Dec 4;30(23):4660. doi: 10.3390/molecules30234660 (PMC12693053; doi:10.3390/molecules30234660)
Supplement: Supplementary file 1 [file molecules-30-04660-s001.zip › molecules-3989950-supplementary.pdf]

molecules

MDPI

Supplementary Materials

Procedural Challenges in Soil Sample Preparation for Pharmaceuticals Analysis

Agnieszka Fiszka Borzyszkowska*, Ewa Olkowska and Lidia Wolska

Department of Environmental Toxicology, Faculty of Health Sciences with Institute of Maritime and Tropical Medicine, Medical University of Gdansk, Debowa 23 A, 80-204 Gdansk, Poland
* Correspondence: agnieszka.fiszka_borzyszkowska@gumed.edu.pl

Table S1. The summary of studies aiming removal of selected pharmaceuticals by ultrasonic (US) technique.

Lp.	Compound	Experimental conditions	Observation	Reference
**1.**	AMOX	C_0 = 100 μM<br>T = 30 °C<br>t = 50 min<br>US power: 300 W	About 20% of AMOX degraded without oxidizing additives.<br>Better removal effect was achieved under acidic conditions.	[1]
		C_0 = 25 mg L^{-1}, 100 mg L^{-1} (in mixture with PARA)<br>t = 180 min<br>US irradiation at 20 kHz and 40 W	About 55 % of AMOX conversion.	[2]
**2.**	AMP	C_0 = 30 μM (10.5 mg L^{-1})<br>t = 30 min<br>optimised ultrasonic system:<br>375 KHz; 24.4 W	100% of AMP degradation under optimised conditions	[3]
**3.**	CIPRO	C_0 = 30 μM<br>t = 120 min<br>ultrasonic power: 100 W<br>pH = 5.0	CIPRO was stable under sonical conditions, other reaction systems were needed, e.g. addition of persulfate or electrooxidation.	[4]
		C_0 = 1 μM,<br>t = 12 min<br>T = 20 °C<br>pH = 8.5<br>sonication power density: 400 W L^{-1}	Sonochemical degradation rates of CIPRO were determined and for primary conditions was equal to 0.22 ± 0.01 μM min^{-1}.<br>The presence terephthalate and fulvic (SRFA) inhibited CIPRO degradation.	[5]
**4.**	ENRO	No data on ENRO ultrasound-assisted degradation. Photochemistry is a significant degradation path for this chemically resistant pollutant.		[6]
**5.**	LINC	No data on LINC ultrasound-assisted degradation. LINC has a very stable structure, due to physicochemical properties, and is hard-degradable.		[7]
**6.**	NIM	No data on NIM ultrasound-assisted degradation		
**7.**	PROP	C_0 = 40 μM<br>t = 30 min<br>ultrasonical frequency: 20 kHz<br>US power: 250 W	The direct sonolysis of PROP was insignificant. The degradation was induced by nano zero-valent iron and/or activated persulfate.	[8]
**8.**	S-ACET	C_0 = 3.31 μM,<br>T = 20 °C<br>t = 30 min<br>pH =7.2	99% of removal under experimental conditions.<br>Detailed examination showed that nitrate, sulphate and chloride did not contribute to the degradation of S-ACET, while bicarbonate accelerated degradation.	[9]

		frequency: 375 kHz<br>US power: 24.4 W<br>in mineral water,<br>in the mixture of 6 pharmaceuticals		
**9.**	S-AMID	No data of S-AMID ultrasound-assisted degradation is available.		
**10.**	TETRA	C_0 = 20 mg L^{-1}<br>the combined action of 20 kHz and 80 kHz ultrasounds<br>t = 60 min	4.8% and 2.0% degradation of TETRA at 20 kHz and 80 kHz, respectively. 14.8% degradation of TETRA under the combined action of 20 kHz and 80 kHz. Degradation process was accelerated by addition of peroxymonosulfate.	[10]
**11.**	TRIM	C_0 = 500 μg L^{-1} or 3 mg L^{-1}<br>pH = 6.0<br>frequency 20 kHz<br>power density = 36 W L^{-1}<br>t = 90 min<br>T = 20.5 ± 0.5 °C	TRIM removal increased with decreasing of initial concentration. After reaction time 73%, 64% and 55% of TRIM degradation was achieved for solutions at 1, 1.5 and 3 mg L^{-1}, respectively.	[11]
**12.**	CARB	C_0 = 0.025 mM<br>US power = 40 W<br>T = 30 °C<br>initial pH = 5.0<br>t = 50 min	CBZ degradation is dependent on: ultrasound power, PS concentration, pH and temperature. Only about 10% was removed in the single ultrasonic process.	[12]
	CARB	C_0 = 0.025 mM;<br>US power: 20 -100 W;<br>200; 400 kHz frequency<br>T = 20 °C ± 3<br>t = 60 min	About 80 % of CARB degradation without pH adjustment under 200 kHz. The degradation rate was directly proportional to ultrasound power.	[13]
**13.**	CAF	C_0 = 5 mg L^{-1}<br>577 kHz frequency<br>US power 120 W<br>T = 25 ± 0.5 °C<br>t = 60 min	CAF was easily decomposed by high-frequency ultrasound. 1-hour of single ultrasonic process allowed for more than 90% of CAF degradation. Other hybrid AOP's were more effective.	[14]
**14.**	MCLO	There is no data on MCLO ultrasound – assisted degradation. MCLO was examined under following stress conditions: oxidative, acid and base hydrolysis, thermal and photolytic to check their stability. MCLO degraded significantly in photolytic, oxidative and thermal conditions and was stable in acid, base, hydrolytic and humidity stress conditions.		[15]
**15.**	MET	There is no data on MET ultrasound – assisted degradation. MET was exposed to UV irradiation, hydrogen peroxide, and ozonation. Photo-induced irradiation supported by hydrogen peroxide was more effective than coupling with ozonation.		[16]
**16.**	PARA	C_0 = 25 mg L^{-1}, 100 mg L^{-1} (in mixture with AMOX)<br>t = 180 min<br>ultrasound irradiation at 20 kHz and 40 W	About 10 % of conversion.	[2]
		C_0 = 3.31 μM<br>T = 20 °C<br>t = 30 min<br>pH = 7.2<br>frequency: 375 kHz<br>power: 24.4 W<br>in mineral water and<br>in the mixture of 6 pharmaceuticals	80% of degradation on distilled water.<br>99 % of degradation in mineral water.	[9]
**17.**	S-CARB	No data of S-CARB ultrasound-assisted degradation is available.		
**18.**	S-DIAZ	C_0 = 25, 50, 70 mg L^{-1}	The percent removals for solutions of 25, 50, and 70 mg L^{-1} were 71.5, 65.7, and 56.0 %, respectively.	[17]

		ultrasonic frequencies: 580, 862, 1,142 kHz<br>t=120 min<br>T=30 °C	The highest SDZ removal was obtained at the lower frequency.<br>The first order constant at pH=5.5 was the highest.	
		C_0= 45 mg L^{-1}<br>pH = 11<br>US power: 200 W m^{-2}<br>t = 80 min	The ultrasound-assisted degradation allowed for 16.3% of S-DIAZ reduction.<br>In other processes observed adsorption capacity on nanostructures materials, e.g. carbon nanotubes and hexagonal MgO. The coupling of all processes with photocatalysis caused improvement of S-DIAZ degradation.	[18]
**19.**	S-GUA	No data of S-GUA ultrasound-assisted degradation is available.		
**20.**	S-MERA	C_0 = 60 mg L^{-1} (0.227 mM)<br>22 kHz frequency<br>power density: 750 W L^{-1}<br>T = 25 ± 2 °C<br>t = 60 min<br>pH = 5.6	With the increase of ultrasonic power to 250 W, the extent of S-MERA degradation was improved up to 23.23 % for 22 + 44 kHz frequency operations. The efficiency was also improved by coupling with UV irradiation.	[19]
**21.**	S-META	C_0 = 9 μM<br>frequency: 800 kHz<br>power: 100 W<br>t = 60 min<br>T = 20 ± 1 °C	In the optimised conditions 94.9% of S-META was degraded.<br>The S-META degradation was inhibited in the presence of nitrates, chlorides, and sulphates and accelerated in the presence of bicarbonates, bromides and ferrous ions.	[20]
	S-META	C_0 = 5 mg L^{-1}<br>t = 60 min<br>frequency: 40 kHz<br>power density: 400 W L^{-1}<br>pH = 3.06 ± 0.2	Less than 10% of S-META was degraded without any additives.<br>S-META degradation was supported by persulfate and nanosized zerovalent copper.	[21]
**22.**	S-THIAZOL	No data of S-THIAZOL ultrasound-assisted degradation is available.		
**23.**	S-ZOL	C_0 = 100 mg L^{-1}<br>ultrasound power density: 600 W L^{-1},<br>pH = 7<br>t = 5 min	Only about 3% of S-ZOL degradation occurs in the presence of ultrasound alone. Coupling of ultrasounds with the ozone oxidation process improved the degradation significantly, more than 95% was degraded within reaction time.	[22]
**24.**	SA	C_0 = 10 mg L^{-1} (in the mixture with other compound: methyl paraben)<br>pH = 3.0<br>t = 30 min<br>sonolysis frequency 572 kHz	About 80% of conversion. Reaction of degradation was pseudo first order, with rate constants of 0.067 min^{-1}	[23]

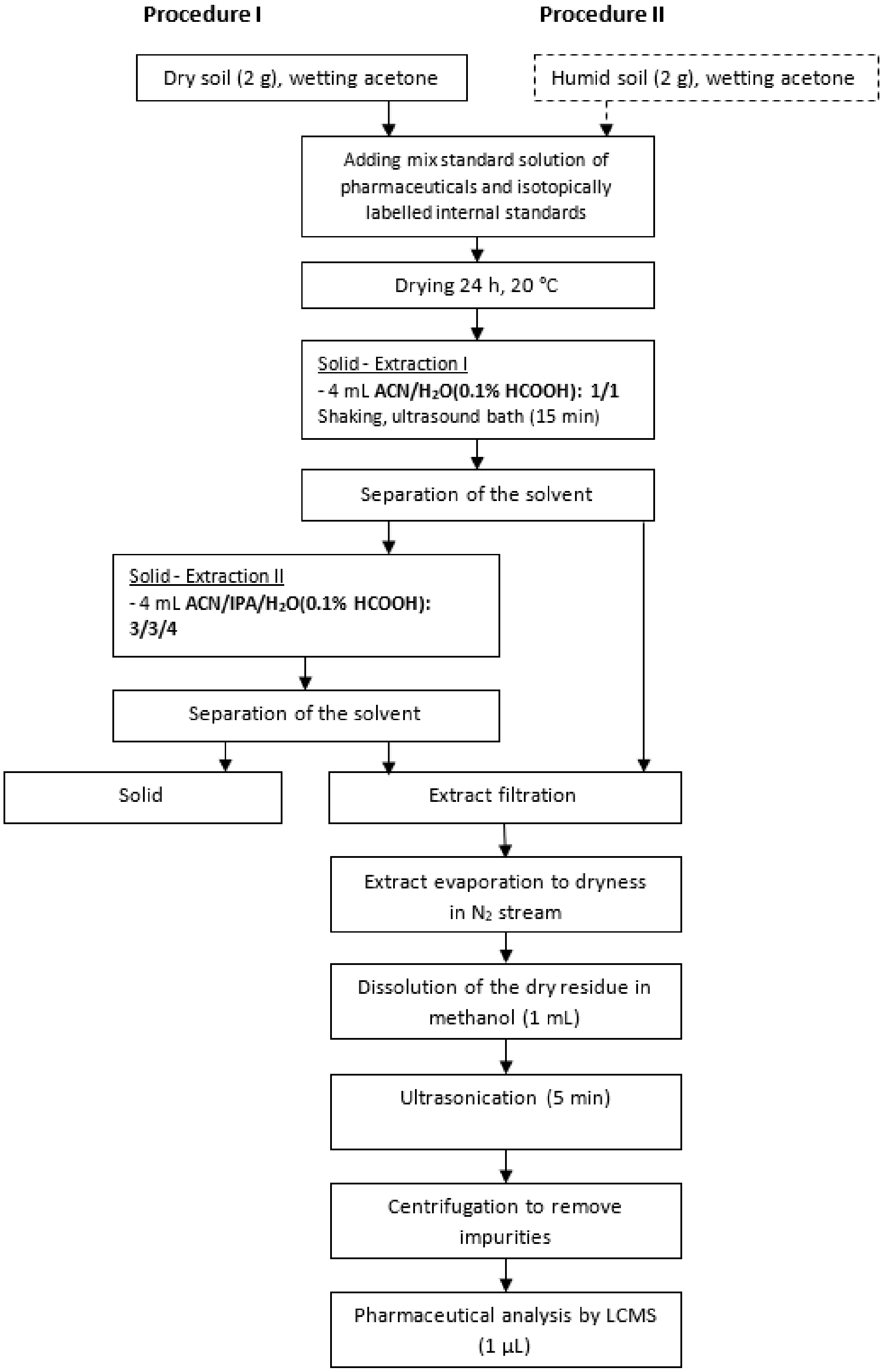

Figure S1. Scheme of procedures no. I-II for determining pharmaceutical analytes in soil samples.

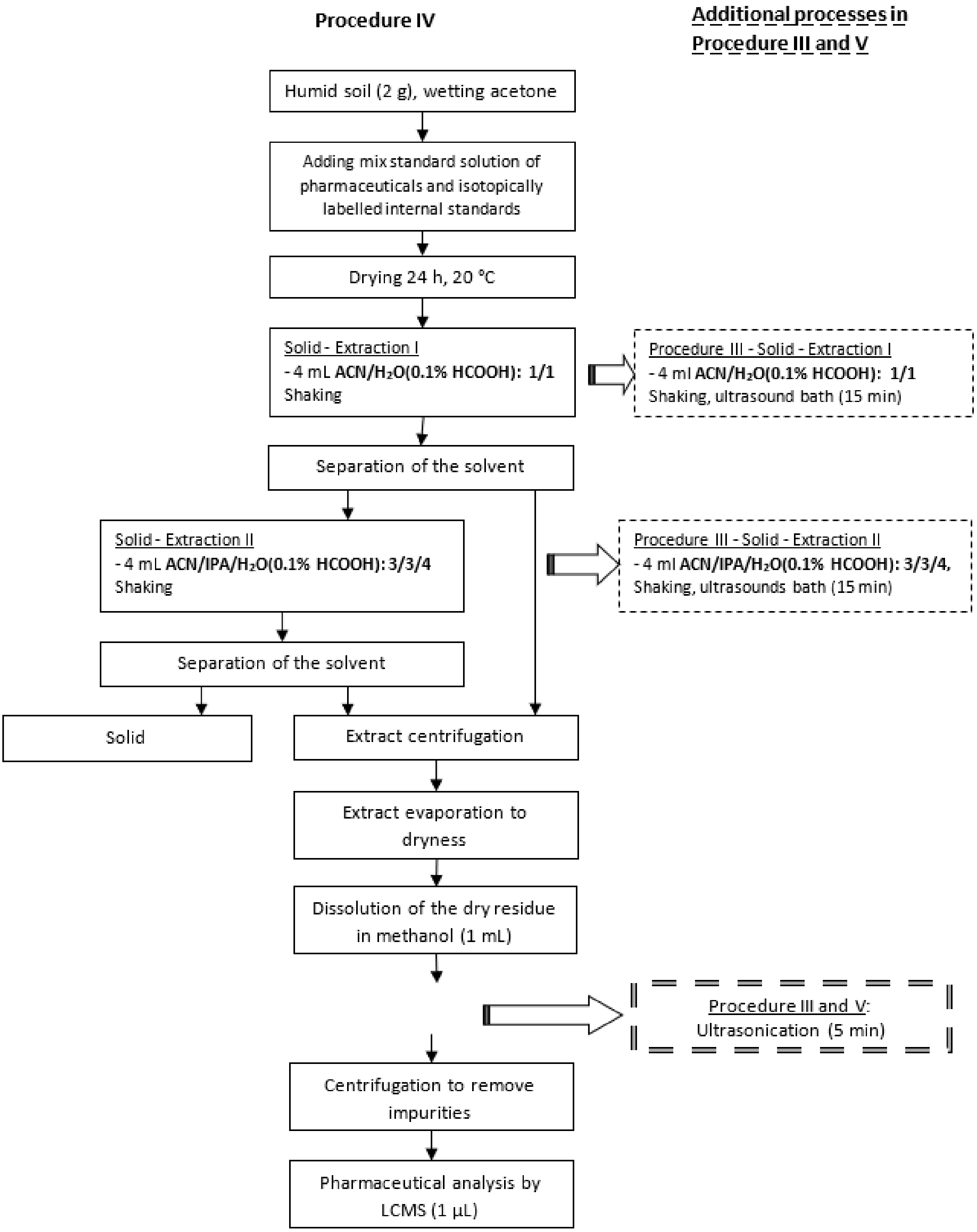

Figure S2. Scheme of procedures no. III-V for determining pharmaceutical analytes in soil samples.

Table S2. Optimized LC-MS/MS instrumental parameters for all analytes, including the isotopically labelled internal standards.

Analyte	Phase mode	Ionisation mode	Precursor ion [m/z]	Product ion [m/z]	Collision energy CE [V]	Q1 pre-rod bias voltage [V]	Q1 pre-rod bias voltage [V]	Retention time [min]
AMOX	acidic	positive	365.90	349.20	-10	-19	-20	2.3
AMP	acidic	positive	350.30	114.00	-35	-25	-28	5.4
AMP-d5	acidic	positive	355.00	160.10	-18	-17	-30	5.4
CIPRO	acidic	positive	332.30	314.20	-22	-12	-19	6.1
CIPRO-d8	acidic	positive	340.25	235.10	-39	-17	-14	6.1
ENRO	acidic	positive	360.20	342.20	-24	-24	-15	6.4
ENRO-d5	acidic	positive	364.95	321.10	-21	-17	-20	6.4
LINC	acidic	positive	407.40	126.20	-31	-10	-25	4.6
MCLO	acidic	positive	300.00	184.10	-33	-30	-30	6
MCLO-d3	acidic	positive	303.20	230.10	-20	-11	-13	6
NIM	acidic	positive	307.00	229.10	17	12	10	8.6
PROP	acidic	positive	260.00	116.20	-21	-30	-29	7.2
S-ACET	acidic	positive	214.90	156.00	-13	-27	-30	2.8
S-AMID	acidic	positive	172.90	92.10	-19	-23	-23	1.3
TETRA	acidic	positive	445.05	410.20	-22	-25	-26	6.1
TETRA-d6	acidic	positive	451.05	416.20	-21	-13	-13	6.1
TRIM	acidic	positive	291.20	261.20	-26	-30	-27	5.4
TRIM-d3	acidic	positive	294.00	123.20	-25	-30	-29	5.4
CARB	alkaline	positive	237.00	194.10	-22	-12	-30	7
CARB-d8	alkaline	positive	245.15	202.20	-22	-19	-29	7
CAF	alkaline	positive	195.00	138.10	-22	-10	-30	5.9
CAF-d9	alkaline	positive	204.05	144.20	-20	-22	-29	5.9
MCLO	alkaline	positive	300.05	184.10	-33	-12	-15	6.6
MCLO-d3	alkaline	positive	303.10	230.10	-21	-11	-13	6.6
MET	alkaline	positive	268.00	116.20	-19	-30	-27	6.8
MET-d7	alkaline	positive	275.05	123.10	-21	-29	-22	6.8
PARA	alkaline	positive	152.10	110.10	-20	-30	-27	2.9
S-ACET	alkaline	positive	215.10	156.10	-12	-15	-15	1.5
S-AMID	alkaline	positive	172.90	91.90	-21	-20	-15	1.6
S-CARB	alkaline	positive	216.00	156.00	-13	-12	-21	0.8
S-DIAZ	alkaline	positive	250.90	156.00	-17	-27	-28	2.1
S-GUA	alkaline	positive	214.90	65.10	-40	-25	-26	1.5
S-MERA	alkaline	positive	264.90	92.10	-29	-13	-14	5.1
S-META	alkaline	positive	279.00	92.10	-32	-14	-21	5.9
S-META-d4	alkaline	positive	283.15	186.10	-19	-20	-30	5.9
S-THIAZOL	alkaline	positive	255.90	92.00	-27	-13	-13	5.2
S-ZOL	alkaline	positive	254.10	92.00	-27	-10	-13	3.6
S-ZOL-d4	alkaline	positive	257.90	96.10	-28	-13	-22	3.6
SA	alkaline	negative	137.30	93.50	15	16	16	2

Table S3. The list of analysed pharmaceuticals and their selected physico-chemical properties.

Lp.	Compound	Molecular formula	Structure	Molecular weight [g/mol]	pKa	LogP	Water solubility
**1.**	AMOX	$C_{16}H_{19}N_3O_5S$	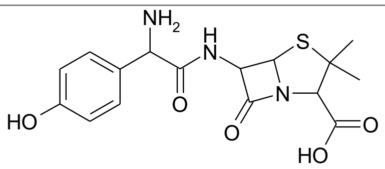	365.4	2.6	0.87	10.7 mg L^{-1}
**2.**	AMP	$C_{16}H_{19}N_3O_4S$	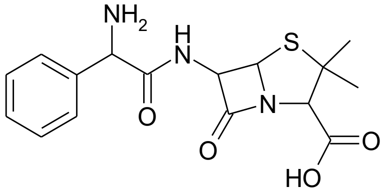	349.4	2.5, 7.3 at 23 °C	1.35	10.1 g L^{-1} (at 21 °C)
**3.**	CIPRO	$C_{17}H_{18}FN_3O_3$	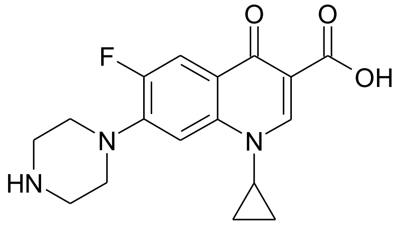	331.3	6.09, 8.74	0.28	30 g L^{-1} (at 20 °C)
**4.**	ENRO	$C_{19}H_{22}FN_3O_3$	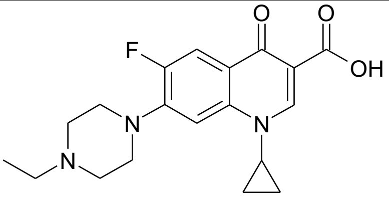	359.4	5.88 - 6.06, 7.70 - 7.74 [24]	0.83 [25]	>53.9 mg L^{-1}
**5.**	LINC	$C_{18}H_{34}N_2O_6S$	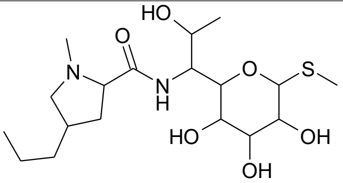	406.5	7.8	0.56	50 g L^{-1} [26]
**6.**	NIM	$C_{13}H_{12}N_2O_5S$	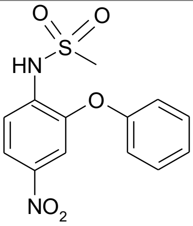	308.31	6.7 [27]	2.6	<0.02 g L^{-1} [28]
**7.**	PROP	$C_{16}H_{21}NO_2$	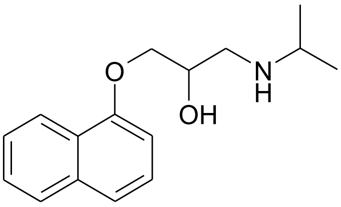	259.34	9.45	3.48	61.7 mg L^{-1}
**8.**	S-ACET	$C_8H_{10}N_2O_3S$	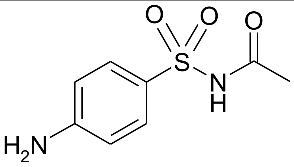	214.24	1.76, 5.22 [29]	-0.96	>32.1 mg L^{-1}

**9.**	S-AMID	$C_6H_8N_2O_2S$	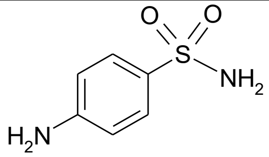	172.21	10.6	-0.62	>25.8 mg L^{-1}
**10.**	TETRA	$C_{22}H_{24}N_2O_8$	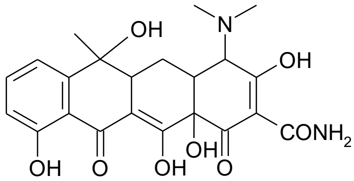	444.4	3.3	-1.3	231 mg L^{-1}
**11.**	TRIM	$C_{14}H_{18}N_4O_3$	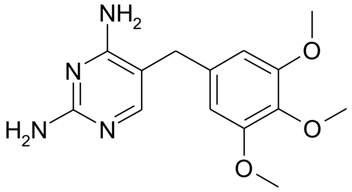	290.32	7.12	0.91	400 mg L^{-1}
**12.**	CARB	$C15H_{12}N_2O$	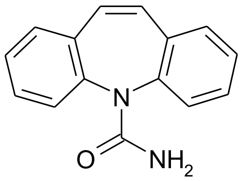	236.27	13.9	2.77	>35.4 mg L^{-1}
**13.**	CAF	$C_8H_{10}N_4O_2$	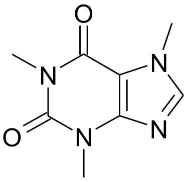	194.19	14	-0.07	21.6 g L^{-1} (at 25 °C)
**14.**	MCLO	$C_{14}H_{22}ClN_3O_2$	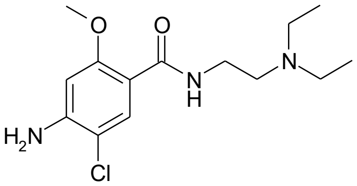	299.79	9.33	2.667	2 mg L^{-1} (at 25 °C)
**15.**	MET	$C_{15}H_{25}NO_3$	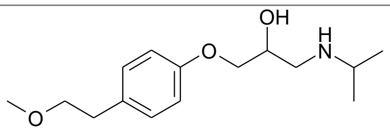	267.36	9.56	1.88	>1000 g L^{-1} (at 25 °C)
**16.**	PARA	$C_8H_9NO_2$	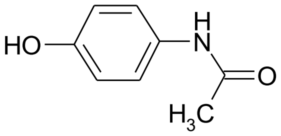	151.16	9.38	0.46	>22.7 mg L^{-1}
**17.**	S-CARB	$C_7H_9N_3O_3S$	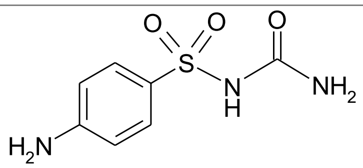	215.23	5.42, 12.22	n.d.	2.33 g L^{-1} (20 °C) [30]
**18.**	S-DIAZ	$C_{10}H_{10}N_4O_2S$	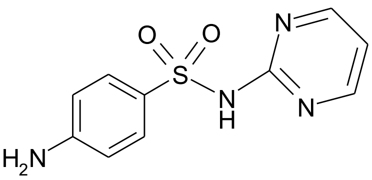	250.28	6.36	-0.09	77 mg L^{-1} (at 25 °C)

**19.**	S-GUA	$C_7H_{10}N_4O_2S$	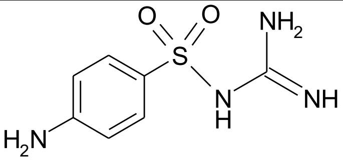	214.25	10.53, 7.72 (predicted) [31]	-0.559 [32]	>32.1 mg L^{-1}
**20.**	S-MERA	$C_{11}H_{12}N_4O_2S$	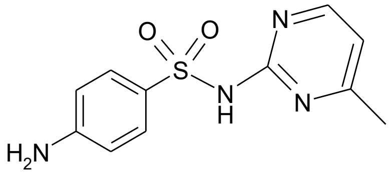	264.31	7.06, 11.92	0.14	202 mg L^{-1} (at 20 °C)
**21.**	S-META	$C_{12}H_{14}N_4O_2S$	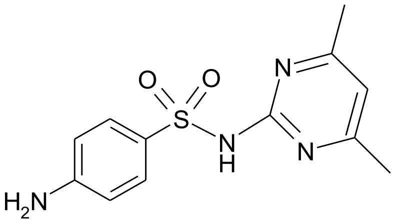	278.33	2.07, 7.49	0.89	1500 mg L^{-1} (at 29 °C)
**22.**	S-THIAZOL	$C_9H_9N_3O_2S_2$	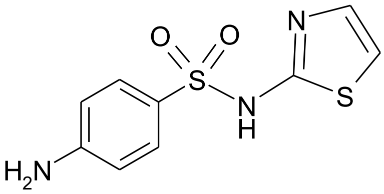	255.3	2.2, 7.24	0.05	373 mg L^{-1} (at 25 °C)
**23.**	S-ZOL	$C_{10}H_{11}N_3O_3S$	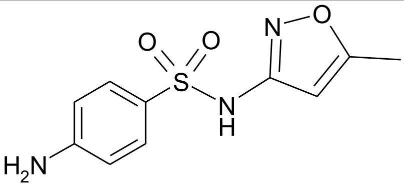	253.28	1.6, 5.7	0.89	>38 mg L^{-1}
**24.**	SA	$C_7H_6O_3$	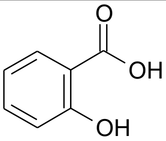	138.12	2.78	2.26	2240 mg L^{-1} (at 25 °C)

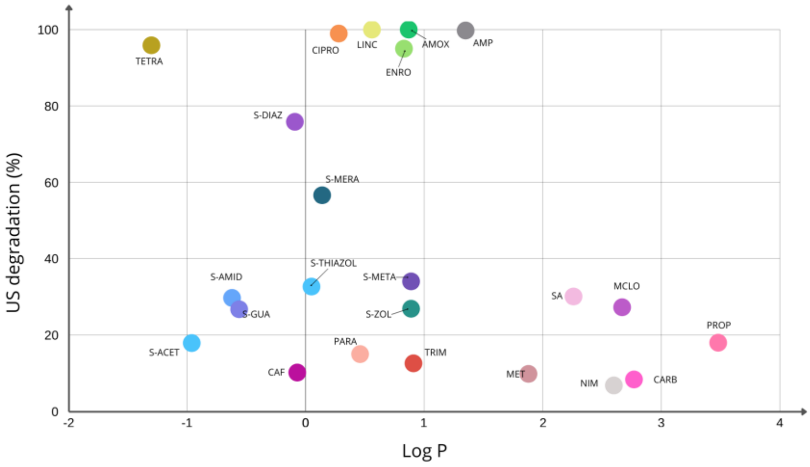

(**a**)

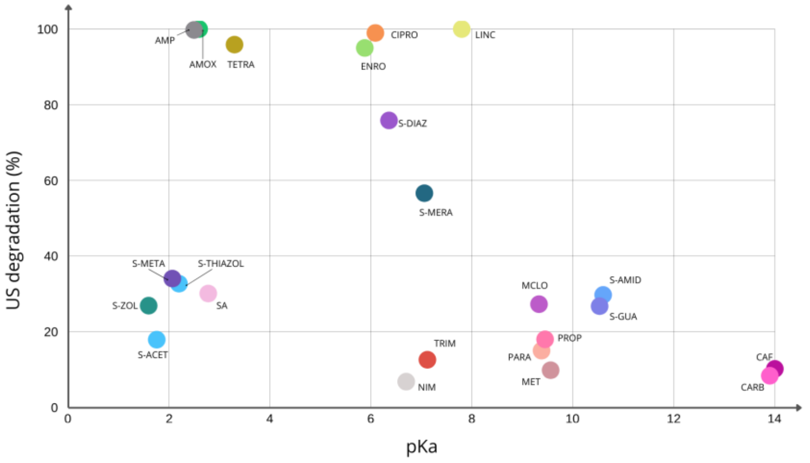

(**b**)

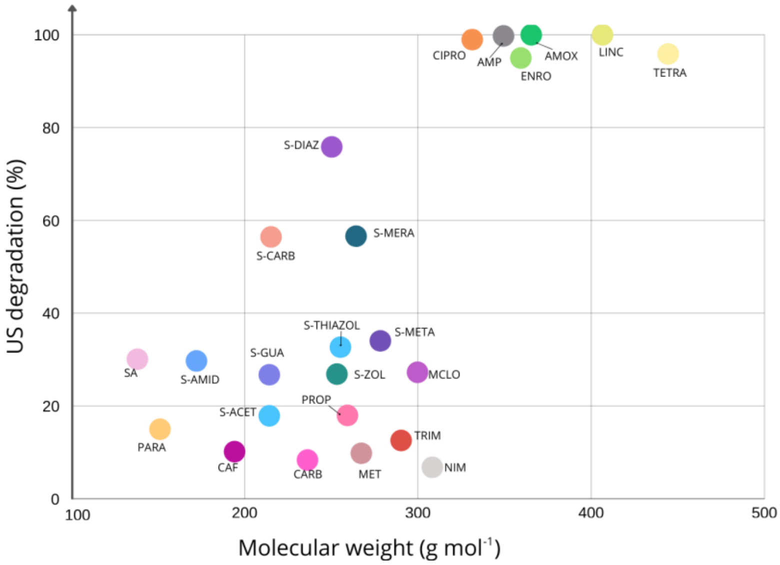

(**c**)

Figure S3. The comparison of the pharmaceuticals' degradation by ultrasound treatment (US) with LogP values (a), pKa values (b) and molecular weight (c).

Table S4. Performance comparison of *p*-values between studied procedures (Procedure I-V) with the *t-tests* for each examined compound.

p-values for procedures (I-V)

very significant difference — less significant difference →

p-value < 0.05

Diagrams - recovery of separated analytes

AMOX	I	II	III	IV	V
I		0.452	0.898	0.538	0.506
II	0.452		0.172	0.602	0.644
III	0.898	0.172		0.169	0.155
IV	0.538	0.602	0.169		0.865
V	0.506	0.644	0.155	0.865	

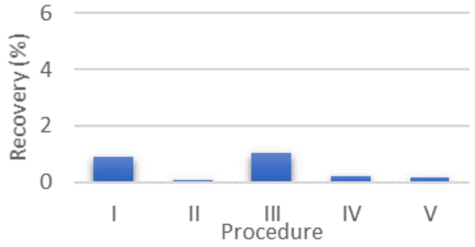

AMP	I	II	III	IV	V
I		0.353	0.559	0.320	0.737
II	0.353		0.787	0.815	0.189
III	0.559	0.787		0.713	0.362
IV	0.320	0.815	0.713		0.123
V	0.737	0.189	0.362	0.123	

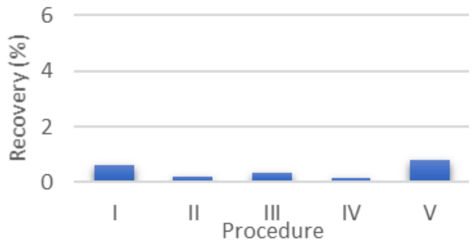

AMP-d5	I	II	III	IV	V
I		0.934	0.149	0.079	0.097
II	0.934		0.210	0.127	0.153
III	0.149	0.210		0.527	0.703
IV	0.079	0.127	0.527		0.121
V	0.097	0.153	0.703	0.121	

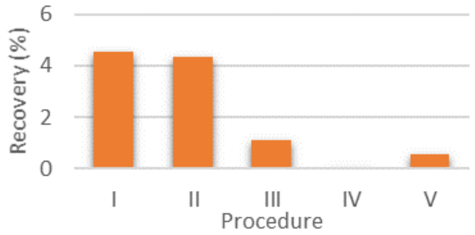

CIPRO	I	II	III	IV	V
I		0.174	0.291	**0.021**	0.382
II	0.174		0.233	**0.001**	0.281
III	0.291	0.233		0.919	0.764
IV	**0.021**	**0.001**	0.919		0.648
V	0.382	0.281	0.764	0.648	

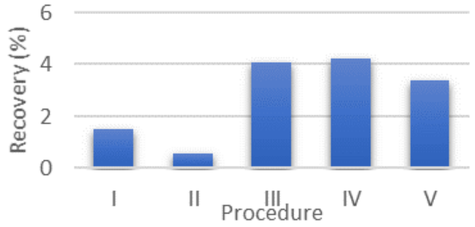

CIPRO-d8	I	II	III	IV	V
I		0.261	**0.001**	0.229	**0.021**
II	0.261		**0.011**	0.211	0.055
III	**0.001**	**0.011**		0.278	0.137
IV	0.229	0.211	0.278		0.578
V	**0.021**	0.055	0.137	0.578	

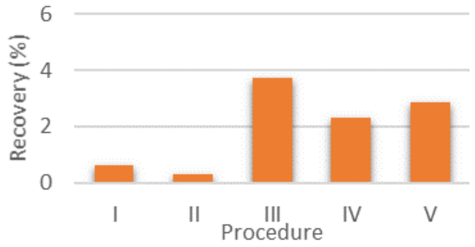

ENRO	I	II	III	IV	V
I		**0.014**	0.942	0.836	0.593
II	**0.014**		0.146	**0.043**	0.076
III	0.942	0.146		0.858	0.785
IV	0.836	**0.043**	0.858		0.476
V	0.593	0.076	0.785	0.476	

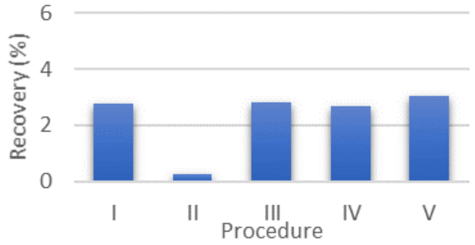

**ENRO-d5**	I	II	III	IV	V
I		0.096	0.247	**0.044**	0.088
II	0.096		0.228	0.051	0.086
III	0.247	0.228		0.827	0.784
IV	**0.044**	0.051	0.827		0.354
V	0.088	0.086	0.784	0.354	

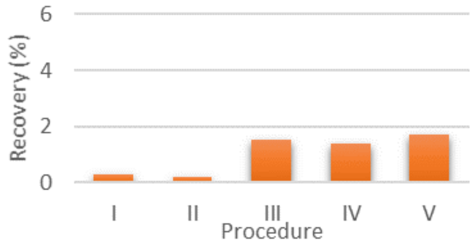

**LINC**	I	II	III	IV	V
I		0.886	0.327	0.133	0.136
II	0.886		0.365	0.231	0.236
III	0.327	0.365		0.633	0.652
IV	0.133	0.231	0.633		0.862
V	0.136	0.236	0.652	0.862	

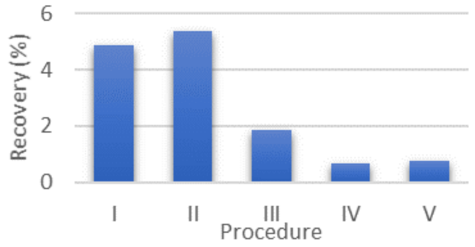

**NIM**	I	II	III	IV	V
I		**0.001**	**0.001**	**0.006**	**0.001**
II	**0.001**		**0.048**	0.647	0.522
III	**0.001**	**0.048**		0.102	0.075
IV	**0.006**	0.647	0.102		0.878
V	**0.001**	0.522	0.075	0.878	

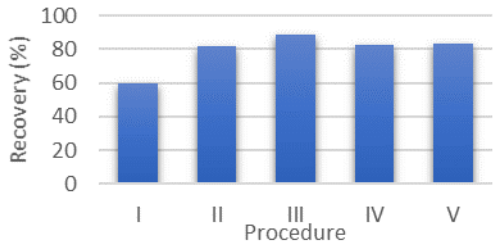

**PROP**	I	II	III	IV	V
I		0.592	0.603	0.729	0.228
II	0.592		0.773	0.745	0.339
III	0.603	0.773		0.701	0.753
IV	0.729	0.745	0.701		0.298
V	0.228	0.339	0.753	0.298	

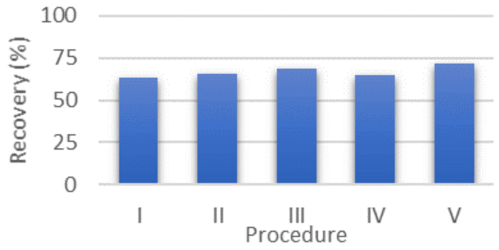

**PROP-d7**	I	II	III	IV	V
I		0.648	0.546	0.745	0.284
II	0.648		0.690	0.918	0.384
III	0.546	0.690		0.663	0.672
IV	0.745	0.918	0.663		0.360
V	0.284	0.384	0.672	0.360	

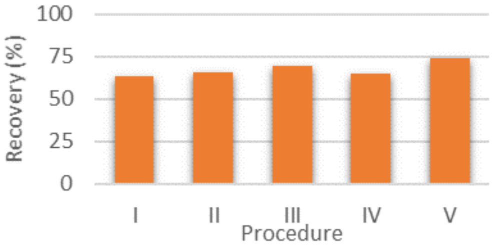

**S-ACET**	I	II	III	IV	V
I		**0.005**	0.557	0.231	0.942
II	**0.005**		0.899	0.244	0.082
III	0.557	0.899		0.704	0.552
IV	0.231	0.244	0.704		0.237
V	0.942	0.082	0.552	0.237	

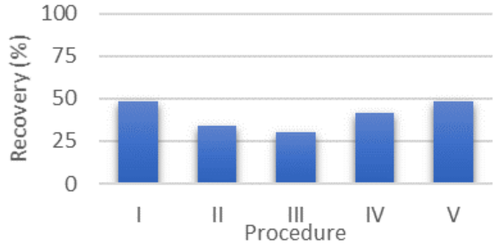

S-AMID	I	II	III	IV	V
I		0.367	0.840	0.079	**0.009**
II	0.367		0.979	0.150	**0.003**
III	0.840	0.979		0.590	0.422
IV	0.079	0.150	0.590		0.267
V	**0.009**	**0.003**	0.422	0.267	

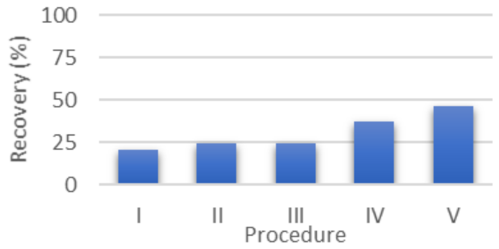

TETRA	I	II	III	IV	V
I		0.194	0.852	0.187	0.954
II	0.194		0.590	0.988	**0.036**
III	0.852	0.590		0.589	0.841
IV	0.187	0.988	0.589		0.066
V	0.954	**0.036**	0.841	0.066	

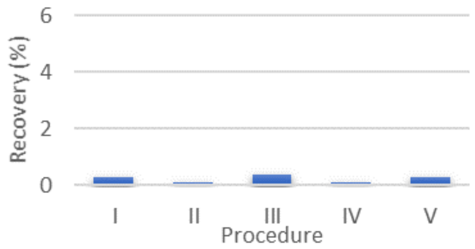

TETRA-d6	I	II	III	IV	V
I		0.083	0.162	0.099	0.777
II	0.083		0.540	0.167	0.337
III	0.162	0.540		0.706	0.426
IV	0.099	0.167	0.706		0.375
V	0.777	0.337	0.426	0.375	

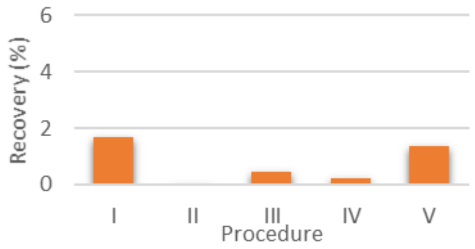

TRIM	I	II	III	IV	V
I		0.854	0.636	0.196	0.830
II	0.854		0.616	0.127	0.918
III	0.636	0.616		0.869	0.600
IV	0.196	0.127	0.869		0.253
V	0.830	0.918	0.600	0.253	

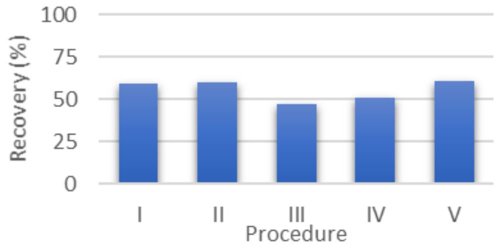

TRIM-d3	I	II	III	IV	V
I		0.910	0.580	0.144	0.906
II	0.910		0.572	0.148	0.830
III	0.580	0.572		0.802	0.596
IV	0.144	0.148	0.802		0.302
V	0.906	0.830	0.596	0.302	

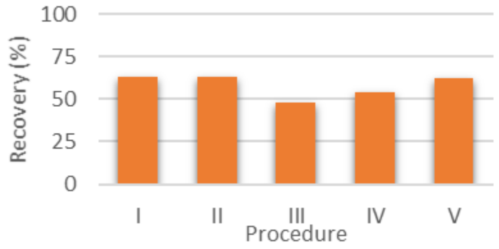

CARB	I	II	III	IV	V
I		0.083	**0.001**	**0.004**	**0.019**
II	0.083		**0.004**	**0.007**	**0.080**
III	**0.001**	**0.004**		**0.027**	0.191
IV	**0.004**	**0.007**	**0.027**		0.497
V	**0.019**	**0.080**	0.191	0.497	

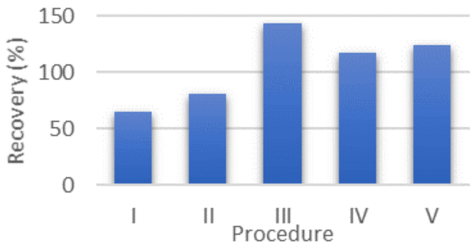

CARB-d8	I	II	III	IV	V
I		0.057	**0.003**	**0.005**	**0.036**
II	0.057		**0.012**	**0.012**	**0.089**
III	**0.003**	**0.012**		0.118	0.936
IV	**0.005**	**0.012**	0.118		0.319
V	**0.036**	**0.089**	0.936	0.319	

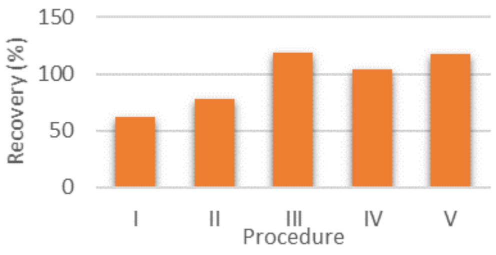

CAF	I	II	III	IV	V
I		0.442	0.144	0.118	0.185
II	0.442		0.173	0.071	0.277
III	0.144	0.173		0.196	0.203
IV	0.118	0.071	0.196		0.420
V	0.185	0.277	0.203	0.420	

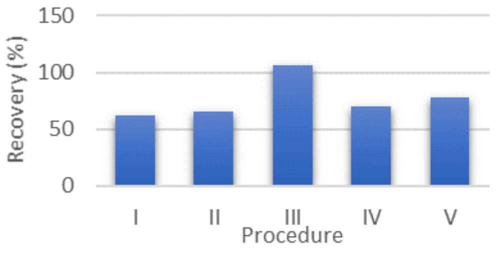

CAF-d9	I	II	III	IV	V
I		0.067	0.096	0.812	0.307
II	0.067		0.200	0.123	0.741
III	0.096	0.200		0.099	0.128
IV	0.812	0.123	0.099		0.275
V	0.307	0.741	0.128	0.275	

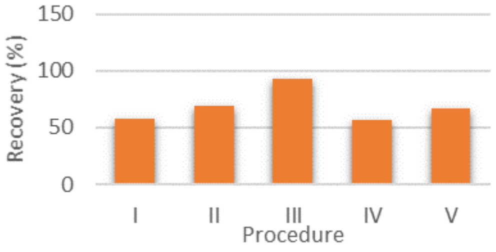

MCLO	I	II	III	IV	V
I		0.901	0.810	**0.022**	**0.050**
II	0.901		0.820	**0.004**	0.122
III	0.810	0.820		0.722	0.667
IV	**0.022**	**0.004**	0.722		0.634
V	**0.050**	0.122	0.667	0.634	

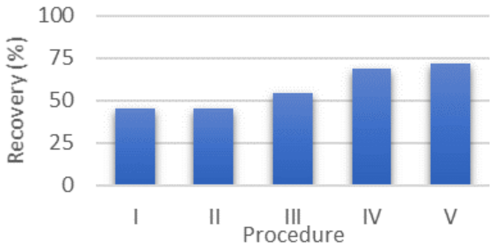

MCLO-d3	I	II	III	IV	V
I		0.587	0.831	**0.026**	**0.025**
II	0.587		0.870	**0.001**	0.094
III	0.831	0.870		0.784	0.686
IV	**0.026**	**0.001**	0.784		0.365
V	**0.025**	0.094	0.686	0.365	

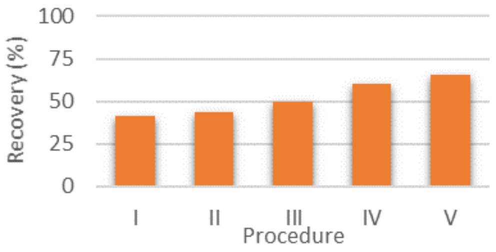

MET	I	II	III	IV	V
I		0.283	**0.044**	**0.018**	**0.006**
II	0.283		0.103	0.082	**0.043**
III	**0.044**	0.103		0.189	0.418
IV	**0.018**	0.082	0.189		0.197
V	**0.006**	**0.043**	0.418	0.197	

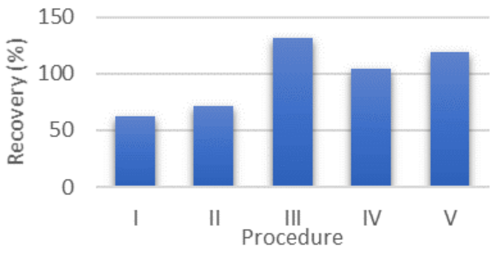

MET-d7	I	II	III	IV	V
I		0.086	**0.014**	**0.006**	**0.019**
II	0.086		0.071	0.076	0.092
III	**0.014**	0.071		0.104	0.247
IV	**0.006**	0.076	0.104		0.322
V	**0.019**	0.092	0.247	0.322	

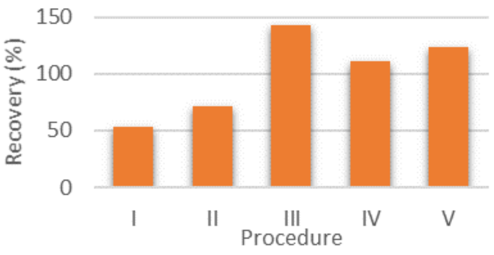

PARA	I	II	III	IV	V
I		**0.002**	0.641	**0.037**	0.340
II	**0.002**		0.234	**0.023**	**0.049**
III	0.641	0.234		0.853	0.849
IV	**0.037**	**0.023**	0.853		0.271
V	0.340	**0.049**	0.849	0.271	

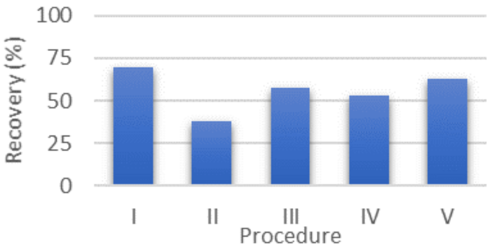

S-CARB	I	II	III	IV	V
I		0.054	0.706	0.062	0.135
II	0.054		0.839	0.061	0.162
III	0.706	0.839		0.727	0.686
IV	0.062	0.061	0.727		0.804
V	0.135	0.162	0.686	0.804	

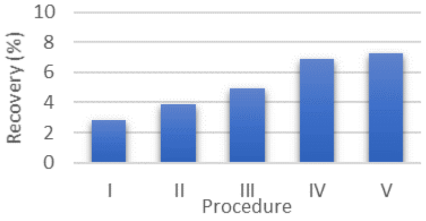

S-DIAZ	I	II	III	IV	V
I		**0.018**	0.876	**0.009**	**0.014**
II	**0.018**		0.674	**0.000**	**0.029**
III	0.876	0.674		0.716	0.626
IV	**0.009**	**0.000**	0.716		0.280
V	**0.014**	**0.029**	0.626	0.280	

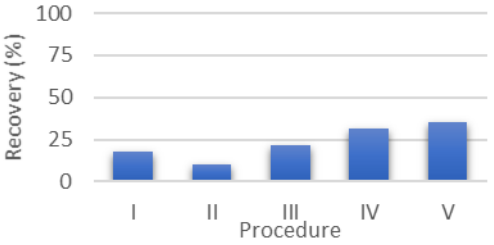

S-GUA	I	II	III	IV	V
I		**0.011**	0.528	**0.026**	**0.050**
II	**0.011**		0.813	**0.003**	**0.037**
III	0.528	0.813		0.688	0.605
IV	**0.026**	**0.003**	0.688		0.373
V	**0.050**	**0.037**	0.605	0.373	

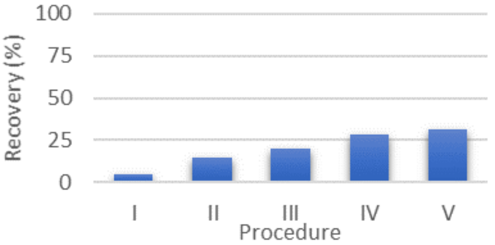

S-MERA	I	II	III	IV	V
I		0.289	0.966	**0.028**	0.085
II	0.289		0.939	**0.002**	0.175
III	0.966	0.939		0.694	0.621
IV	**0.028**	**0.002**	0.694		0.536
V	0.085	0.175	0.621	0.536	

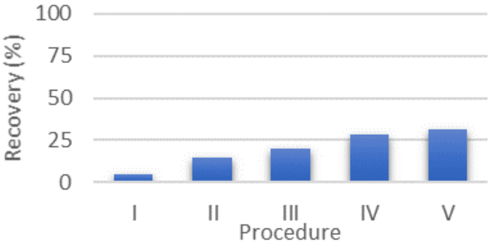

S-META	I	II	III	IV	V
I		0.184	0.731	**0.001**	**0.022**
II	0.184		0.796	**0.001**	**0.050**
III	0.731	0.796		0.741	0.657
IV	**0.001**	**0.001**	0.741		0.342
V	**0.022**	**0.050**	0.657	0.342	

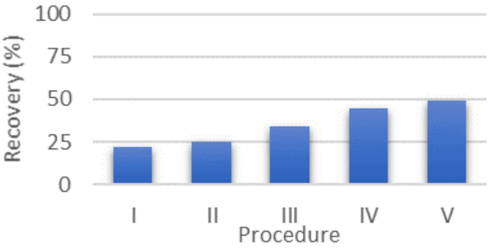

S-META-d4	I	II	III	IV	V
I		0.062	0.735	**0.001**	**0.045**
II	0.062		0.823	**0.012**	**0.079**
III	0.735	0.823		0.735	0.644
IV	**0.001**	**0.012**	0.735		0.355
V	**0.045**	**0.079**	0.644	0.355	

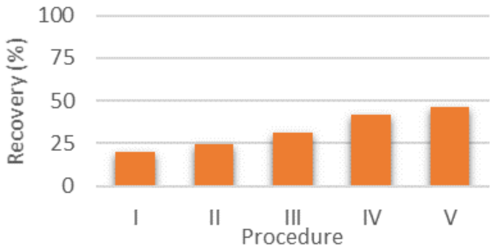

S-THIAZOL	I	II	III	IV	V
I		0.427	0.819	**0.012**	**0.022**
II	0.427		0.881	**0.031**	0.099
III	0.819	0.881		0.738	0.667
IV	**0.012**	**0.031**	0.738		0.451
V	**0.022**	0.099	0.667	0.451	

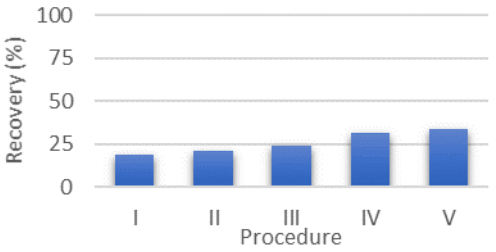

S-ZOL	I	II	III	IV	V
I		**0.002**	0.635	**0.007**	**0.032**
II	**0.002**		0.987	0.085	0.147
III	0.635	0.987		0.776	0.728
IV	**0.007**	0.085	0.776		0.642
V	**0.032**	0.147	0.728	0.642	

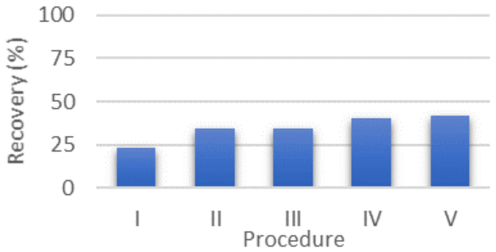

S-ZOL-d4	I	II	III	IV	V
I		**0.010**	0.663	**0.006**	**0.036**
II	**0.010**		0.971	**0.019**	0.191
III	0.663	0.971		0.788	0.719
IV	**0.006**	**0.019**	0.788		0.562
V	**0.036**	0.191	0.719	0.562	

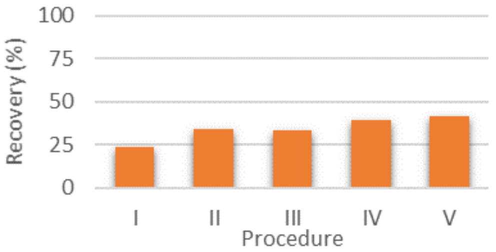

SA	I	II	III	IV	V
I		0.310	0.888	0.286	0.962
II	0.310		0.992	0.238	0.906
III	0.888	0.992		0.634	0.936
IV	0.286	0.238	0.634		0.668
V	0.962	0.906	0.936	0.668	

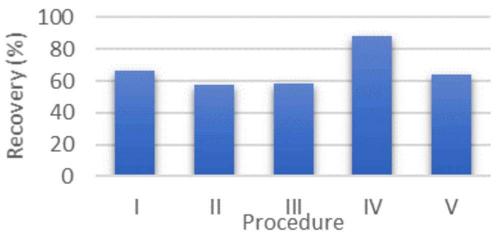

References

1. Sun, X.; Qin, Y.; Zhou, W. Degradation of Amoxicillin from Water by Ultrasound-Zero-Valent Iron Activated Sodium Persulfate. *Sep. Purif. Technol.* **2021**, *275*, 119080, doi:10.1016/j.seppur.2021.119080.
2. Stucchi, M.; Rigamonti, M.G.; Carnevali, D.; Boffito, D.C. A Kinetic Study on the Degradation of Acetaminophen and Amoxicillin in Water by Ultrasound. *ChemistrySelect* **2020**, *5*, 14986–14992, doi:10.1002/slct.202004147.
3. Montoya-Rodríguez, D.M.; Serna-Galvis, E.A.; Ferraro, F.; Torres-Palma, R.A. Degradation of the Emerging Concern Pollutant Ampicillin in Aqueous Media by Sonochemical Advanced Oxidation Processes - Parameters Effect, Removal of Antimicrobial Activity and Pollutant Treatment in Hydrolyzed Urine. *J. Environ. Manage.* **2020**, *261*, doi:10.1016/j.jenvman.2020.110224.
4. Ma, X.; Wang, Z. Removal of Ciprofloxacin from Wastewater by Ultrasound/Electric Field/Sodium Persulfate (US/E/PS). *Processes* **2022**, *10*, doi:10.3390/pr10010124.
5. Xiao, R.; He, Z.; Diaz-Rivera, D.; Pee, G.Y.; Weavers, L.K. Sonochemical Degradation of Ciprofloxacin and Ibuprofen in the Presence of Matrix Organic Compounds. *Ultrason. Sonochem.* **2014**, *21*, doi:10.1016/j.ultsonch.2013.06.012.

6. Sturini, M.; Speltini, A.; Maraschi, F.; Profumo, A.; Pretali, L.; Fasani, E.; Albini, A. Sunlight-Induced Degradation of Soil-Adsorbed Veterinary Antimicrobials Marbofloxacin and Enrofloxacin. *Chemosphere* **2012**, *86*, doi:10.1016/j.chemosphere.2011.09.053.
7. Zhang, L.; Shen, Y.; Hui, F.; Niu, Q. Degradation of Residual Lincomycin in Fermentation Dregs by Yeast Strain S9 Identified as Galactomyces Geotrichum. *Ann. Microbiol.* **2015**, *65*, doi:10.1007/s13213-014-0971-3.
8. Gao, Y. qiong; Gao, N. yun; Wang, W.; Kang, S. fei; Xu, J. hong; Xiang, H. ming; Yin, D. qiang Ultrasound-Assisted Heterogeneous Activation of Persulfate by Nano Zero-Valent Iron (NZVI) for the Propranolol Degradation in Water. *Ultrason. Sonochem.* **2018**, *49*, doi:10.1016/j.ultsonch.2018.07.001.
9. Camargo-Perea, A.L.; Serna-Galvis, E.A.; Lee, J.; Torres-Palma, R.A. Understanding the Effects of Mineral Water Matrix on Degradation of Several Pharmaceuticals by Ultrasound: Influence of Chemical Structure and Concentration of the Pollutants. *Ultrason. Sonochem.* **2021**, *73*, doi:10.1016/j.ultsonch.2021.105500.
10. Zeng, L.; Huang, C.; Tang, Y.; Wang, C.; Lin, S. Tetracycline Degradation by Dual-Frequency Ultrasound Combined with Peroxymonosulfate. *Ultrason. Sonochem.* **2024**, *106*, 106886, doi:10.1016/j.ultsonch.2024.106886.
11. Arvaniti, O.S.; Frontistis, Z.; Nika, M.C.; Aalizadeh, R.; Thomaidis, N.S.; Mantzavinos, D. Sonochemical Degradation of Trimethoprim in Water Matrices: Effect of Operating Conditions, Identification of Transformation Products and Toxicity Assessment. *Ultrason. Sonochem.* **2020**, *67*, doi:10.1016/j.ultsonch.2020.105139.
12. Huang, X.; Wang, Z.; Sun, Z.; Wang, Z. Degradation of Carbamazepine from Wastewater by Ultrasound-Enhanced Zero-Valent Iron -Activated Persulfate System (US/Fe0/PS): Kinetics, Intermediates and Pathways. *Environ. Technol. (United Kingdom)* **2024**, *45*, 1760–1769, doi:10.1080/09593330.2022.2152737.
13. Rao, Y.; Yang, H.; Xue, D.; Guo, Y.; Qi, F.; Ma, J. Sonolytic and Sonophotolytic Degradation of Carbamazepine: Kinetic and Mechanisms. *Ultrason. Sonochem.* **2016**, *32*, doi:10.1016/j.ultsonch.2016.04.005.
14. Ziylan-Yavas, A.; Ince, N.H.; Ozon, E.; Arslan, E.; Aviyente, V.; Savun-Hekimoğlu, B.; Erdincler, A. Oxidative Decomposition and Mineralization of Caffeine by Advanced Oxidation Processes: The Effect of Hybridization. *Ultrason. Sonochem.* **2021**, *76*, doi:10.1016/j.ultsonch.2021.105635.
15. Sowjanya, P.; Shanmugasundaram, P.; Naidu, P.; Singamsetty, S.K. Novel Validated Stability-Indicating UPLC Method for the Determination of Metoclopramide and Its Degradation Impurities in API and Pharmaceutical Dosage Form. *J. Pharm. Res.* **2013**, *6*, doi:10.1016/j.jopr.2013.07.004.
16. Voigt, M.; Bartels, I.; Schmiemann, D.; Votel, L.; Hoffmann-Jacobsen, K.; Jaeger, M. Metoprolol and Its Degradation and Transformation Products Using Aops-Assessment of Aquatic Ecotoxicity Using Qsar. *Molecules* **2021**, *26*, doi:10.3390/molecules26113102.
17. Lastre-Acosta, A.M.; Cruz-González, G.; Nuevas-Paz, L.; Jáuregui-Haza, U.J.; Teixeira, A.C.S.C. Ultrasonic Degradation of Sulfadiazine in Aqueous Solutions. *Environ. Sci. Pollut. Res.* **2015**, *22*, doi:10.1007/s11356-014-2766-2.
18. Hayati, F.; Isari, A.A.; Anvaripour, B.; Fattahi, M.; Kakavandi, B. Ultrasound-Assisted Photocatalytic Degradation of Sulfadiazine Using MgO@CNT Heterojunction Composite: Effective Factors, Pathway and Biodegradability Studies. *Chem. Eng. J.* **2020**, *381*, doi:10.1016/j.cej.2019.122636.
19. Agarkoti, C.; Chaturvedi, A.; Gogate, P.R.; Pandit, A.B. Degradation of Sulfamerazine Using Ultrasonic Horn and Pilot Scale US Reactor in Combination with Different Oxidation Approaches. *Sep. Purif. Technol.* **2023**, *312*, 123351, doi:10.1016/j.seppur.2023.123351.
20. Gao, Y.Q.; Gao, N.Y.; Deng, Y.; Gu, J.S.; Gu, Y.L.; Zhang, D. Factors Affecting Sonolytic Degradation of Sulfamethazine in Water. *Ultrason. Sonochem.* **2013**, *20*, doi:10.1016/j.ultsonch.2013.04.007.
21. Zhang, T.; Yang, Y.; Li, X.; Yu, H.; Wang, N.; Li, H.; Du, P.; Jiang, Y.; Fan, X.; Zhou, Z. Degradation of Sulfamethazine by Persulfate Activated with Nanosized Zero-Valent Copper in Combination with Ultrasonic Irradiation. *Sep. Purif. Technol.* **2020**, *239*, doi:10.1016/j.seppur.2020.116537.
22. Guo, W.Q.; Yin, R.L.; Zhou, X.J.; Du, J.S.; Cao, H.O.; Yang, S.S.; Ren, N.Q. Sulfamethoxazole Degradation by Ultrasound/Ozone Oxidation Process in Water: Kinetics, Mechanisms, and Pathways. *Ultrason. Sonochem.* **2015**, *22*, doi:10.1016/j.ultsonch.2014.07.008.
23. Arslan, E.; Hekimoglu, B.S.; Cinar, S.A.; Ince, N.; Aviyente, V. Hydroxyl Radical-Mediated Degradation of Salicylic Acid and Methyl Paraben: An Experimental and Computational Approach to Assess the Reaction Mechanisms. *Environ. Sci. Pollut. Res.* **2019**, *26*, doi:10.1007/s11356-019-06048-3.

24. Barbosa, J.; Barrón, D.; Jiménez-Lozano, E.; Sanz-Nebot, V. Comparison between Capillary Electrophoresis, Liquid Chromatography, Potentiometric and Spectrophotometric Techniques for Evaluation of PKa Values of Zwitterionic Drugs in Acetonitrile-Water Mixtures. *Anal. Chim. Acta* **2001**, *437*, doi:10.1016/S0003-2670(01)00997-7.
25. Van Doorslaer, X.; Dewulf, J.; Van Langenhove, H.; Demeestere, K. Fluoroquinolone Antibiotics: An Emerging Class of Environmental Micropollutants. *Sci. Total Environ.* 2014, *500–501*.
26. Information about Lincomycine from Sigma Aldrich. Avaiable online: https://www.sigmaaldrich.com/PL/pl/product/sigma/l6004?srsltid=AfmBOoogB_UQAzx4ad641ztp-anbT3AaXaDqk0tlCszsNJDFzPjy_d.
27. Pauletto, P.S.; Lütke, S.F.; Dotto, G.L.; Salau, N.P.G. Adsorption Mechanisms of Single and Simultaneous Removal of Pharmaceutical Compounds onto Activated Carbon: Isotherm and Thermodynamic Modeling. *J. Mol. Liq.* **2021**, *336*, doi:10.1016/j.molliq.2021.116203.
28. Wei, W.; Evseenko, V.I.; Khvostov, M. V.; Borisov, S.A.; Tolstikova, T.G.; Polyakov, N.E.; Dushkin, A. V.; Xu, W.; Min, L.; Su, W. Solubility, Permeability, Anti-Inflammatory Action and in Vivo Pharmacokinetic Properties of Several Mechanochemically Obtained Pharmaceutical Solid Dispersions of Nimesulide. *Molecules* **2021**, *26*, doi:10.3390/molecules26061513.
29. Guo, Y.; Liu, Z.; Lou, X.; Fang, C.; Wang, P.; Wu, G.; Guan, J. Insights into Antimicrobial Agent Sulfacetamide Transformation during Chlorination Disinfection Process in Aquaculture Water. *RSC Adv.* **2021**, *11*, doi:10.1039/d1ra01605a.
30. Information about Sulfacarbamide from Chemicalbook. Available online: https://www.chemicalbook.com/ChemicalProductProperty_IN_CB3711796.htm.
31. Information about Sulfacarbamide from Drugback. Available online: https://go.drugbank.com/drugs/DB13726.
32. Information about Sulfacarbamide from Chemeo. Available online: https://www.chemeo.com/cid/12-308-6/Sulfaguanidine.
